# Global late Quaternary megafauna extinctions linked to humans, not climate change

**DOI:** 10.1098/rspb.2013.3254

**Published:** 2014-07-22

**Authors:** Christopher Sandom, Søren Faurby, Brody Sandel, Jens-Christian Svenning

**Affiliations:** Ecoinformatics and Biodiversity, Department of Bioscience, Aarhus University, Ny Munkegade 114, Aarhus C 8000, Denmark

**Keywords:** climate change, macroecology, megafauna extinction, overkill, palaeoecology

## Abstract

The late Quaternary megafauna extinction was a severe global-scale event. Two factors, climate change and modern humans, have received broad support as the primary drivers, but their absolute and relative importance remains controversial. To date, focus has been on the extinction chronology of individual or small groups of species, specific geographical regions or macroscale studies at very coarse geographical and taxonomic resolution, limiting the possibility of adequately testing the proposed hypotheses. We present, to our knowledge, the first global analysis of this extinction based on comprehensive country-level data on the geographical distribution of all large mammal species (more than or equal to 10 kg) that have gone globally or continentally extinct between the beginning of the Last Interglacial at 132 000 years BP and the late Holocene 1000 years BP, testing the relative roles played by glacial–interglacial climate change and humans. We show that the severity of extinction is strongly tied to hominin palaeobiogeography, with at most a weak, Eurasia-specific link to climate change. This first species-level macroscale analysis at relatively high geographical resolution provides strong support for modern humans as the primary driver of the worldwide megafauna losses during the late Quaternary.

## 1. Introduction

During the Late Pleistocene and early Holocene, regions around the world suffered losses of megafauna species of a magnitude unprecedented for many millions of years [1–3]. Although extinctions are common in the Quaternary fossil record, such rapid and large global species losses without functional replacements are unusual. Multiple explanatory hypotheses for this global extinction event have been proposed, including climate change [4,5], the spread of modern humans (*Homo sapiens*) and related effects of hunting and habitat change [6–9], an extra-terrestrial impact [10] and hyper-disease [11]. With little or no evidence to support the latter two as global explanations [11–14], we focus here on climate change and hominin palaeobiogeography. The relative importance of climate and hominin drivers has been subject to long-standing, highly charged controversy (e.g. [3,15,16]).

Although megafaunal extinctions occurred worldwide, most studies focus on a specific region [17–19], and the few global-scale studies have been geographically incomplete and/or very coarse-grained [3,20]. In an influential review, Barnosky *et al*. [3] provided a continental breakdown of the Late Pleistocene megafauna extinctions and reported that humans were the main extinction driver in Australia and North America, and climate was the main driver for Europe, but that data were insufficient for making assessments for Africa, Asia and South America. Nogués-Bravo *et al*. [20] followed up by linking these genus-level continental-resolution patterns to continental-average climate change, finding extinction patterns among Africa, Eurasia and North America consistent with climate-driven extinction; however, South America strongly deviated from this trend, with the lowest climate change and the most severe genus-level extinctions. A geographically comprehensive species-level assessment of the global-scale extinction pattern remains lacking. Improvements and increased access to fossil, palaeoclimate and hominin palaeobiogegraphical records, more accurate dating and taxonomic revisions now make possible much more geographically fine-grained analysis of megafauna extinctions worldwide, namely species-level extinctions per Taxonomic Databases Working Group (TDWG) level 3 country (see Material and methods for a description of these regions).

### (a) The climate change hypothesis

**Figure 1.**
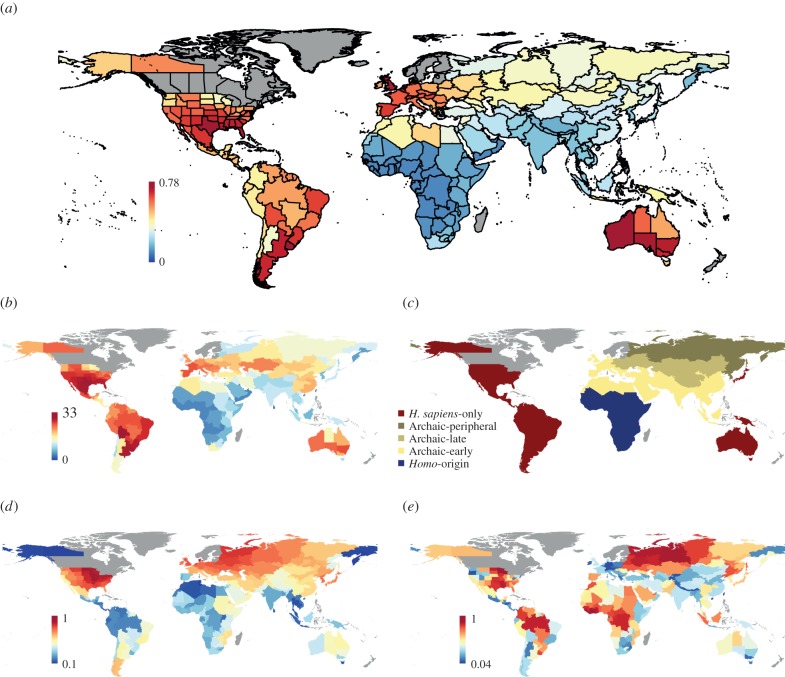
Global maps of late Quaternary large mammal extinction severity, hominin palaeobiogeography, temperature anomaly and precipitation velocity. (*a*) The proportion of extinct large mammal species (more than or equal to 10 kg) in each TDWG country during the last 132 000 years, only counting extinctions earlier than 1000 years BP. (*b*) The cumulative number of extinct large mammal species occurring in each TDWG country. (*c*) Hominin palaeobiogeography (see the text for further explanation). (*d*) Mean anomaly in mean annual temperature between the LGM and today. (*e*) Mean velocity in annual precipitation between the LGM and today. TDWG countries shaded in dark grey were excluded from analyses. The climate change variables were standardized to range between 0 and 1.

We focus on temperature and precipitation contrasts between full glacial and full interglacial conditions, as these represent the strongest climate shifts during the late Quaternary. We estimated these as the Last Glacial Maximum (LGM, *ca* 21 000 years BP) to present-day (1950–2000) contrast and, in supplement, the Last Interglacial (LIG, *ca* 130 000 years BP) to LGM contrast. Palaeoclimatic changes can be represented both by simple macroclimatic changes through time or by climate change velocities that incorporate spatial climate gradients to estimate how fast climate moved in space, providing a direct estimate of how rapidly species would have needed to migrate to track climate [21,22]. Where climate change is severe and the topography is flat, species would typically have to travel greater distances to find suitable conditions, experiencing greater risks of extinction. As a result, high late Quaternary climate change velocity is associated with low modern species endemism in mammals, birds and amphibians worldwide, indicating greater past extinction rates in more climatically unstable regions [21]. We used mean annual temperature and annual precipitation anomaly and velocity between the LGM and the present to represent the potential severity of climate change over the study period (figure 1*d*,*e* and the electronic supplementary material, figure S1). If climate change has been an important driver of extinction, then climate change magnitude or velocity should be positively correlated with the proportion of the cumulative late Quaternary large mammal species per TDWG country that have become globally or continentally extinct during 132 000–1000 years BP. Later extinctions were not considered because of the strong known human effects during this period. Although our analysis is at the country scale and species may respond to more local environments, large mammals generally have large ranges [23] and within-TDWG country variation in climate change velocity is typically small (see Material and methods).

### (b) The human palaeobiogeography hypothesis

Hominins have migrated out of Africa in waves successively increasing the hominin-occupied area so that megafauna have co-occurred and coevolved with hominins for differing lengths of time in different regions. Possibly as early as the Late Pliocene or Early Pleistocene, *Homo erectus* and contemporary hominins expanded into southern Asia and south and western Europe [24] (figure 1*c* ‘Archaic-early’). *Homo neanderthalensis* and Denisovan humans pushed these boundaries further, colonizing most of Eurasia and leaving only the very northern extremities unpopulated during interglacials [25] (figure 1*c* ‘Archaic-late’). While archaic humans appear not to have inhabited the northernmost parts of Eurasia, no terrestrial megafaunal species was endemic to these parts (see the electronic supplementary material, Data S1). The residing megafauna would have encountered archaic hominins during range extensions into more southerly regions or rare archaic incursions into the north; thus, we classify this region with those occupied by archaic hominins in our main analyses (figure 1*c* ‘Archaic-peripheral’). By contrast, faunas in Australia, North and South America and to some extent Japan did not have any contact with hominins until the expansion of modern humans [26] (figure 1*c* ‘*Homo sapiens*-only’). Late Quaternary megafauna extinctions have been hypothesized to have been more severe where modern humans were the first hominin to arrive, suddenly introducing a new and effective big-game predator into regions with megafauna naive to human hunting [27]. Conversely, extinctions have been posited to have been less severe in Africa because of long-term, gradual hominin–megafauna coevolution [3]; alternatively, pre-Late Pleistocene megafauna extinctions in regions occupied by pre-*sapiens* hominins may also have contributed to fewer late Quaternary extinctions [27,28], or human populations may have been more suppressed by disease here [15]. Under this scenario, we hypothesize that the most severe extinctions occurred where modern humans were the first hominins to arrive and that the lowest extinction occurred in sub-Saharan Africa, the centre of origin and evolution for hominins overall as well as for *H. sapiens* [24] (figure 1*c* ‘*Homo*-origin’).

We aim to evaluate the relative support for the climate change and hominin palaeobiogeography hypotheses for the late Quaternary megafauna extinctions by, to our knowledge, the first comprehensive species-level, fine-grained global macroecological analysis. We achieve this aim and find strong support for hominin palaeobiogeography as the critical factor for explaining the strong geographical variation in megafauna extinction severity worldwide.

## 2. Material and methods

### (a) Extinct species of the late Quaternary

A thorough examination of the scientific literature identified 177 taxonomically accepted, extinct or continentally extirpated large mammals (more than or equal to 10 kg) that occurred during the Late Pleistocene and early/middle Holocene (132 000–1000 years BP). The process was begun by gathering information on species names reported lost during the late Quaternary from review publications and original research articles followed by detailed species by species research to confirm their taxonomic validity and presence within our study period (electronic supplementary material, table S1). In some circumstances, we chose to reject uncertain species closely related to accepted species occurring over similar ranges to ensure that our extinction densities were conservative. A notable example includes the North American horses, where only two *Equus* species were accepted, *Equus ferus* and *Equus semiplicatus* (representing the stilt-legged horses), following [29]. Species were only accepted if they are recorded from a directly dated site within the time period studied. In order to be conservative, we did not accept dates based on co-occurrence with potential indicator fossils, i.e. species thought to be associated with a particular time period. As a result, the number of extinctions that are too old to be correctly dated with carbon-14 dating may be underestimated in our analysis. Species that were of uncertain taxonomy or occurred from sites thought to be within the late Quaternary, but lack a confirmed date were included in a list of potential, but uncertain late Quaternary megafauna extinctions (*n* = 27; electronic supplementary material, table S1). Of these 27 taxonomically or temporally uncertain species, we recorded regional presences for 17 of them, which were included in a supporting sensitivity analysis (electronic supplementary material, table S3). Species mass were primarily collected from Smith *et al*. [30] and supplemented with additional sources or, where required, were estimated based on masses of species in the same genus (electronic supplementary material, table S1). We defined ‘large’ mammals as more than or equal to 10 kg (*n* = 177 spp.). We repeated the analyses including species more than or equal to 44 kg (*n* = 154 spp.) to be consistent with much of the literature published on the megafauna extinctions [3]. Species lost in the last 1000 years were excluded because of their much clearer association to human drivers of extinction.

### (b) Extinct species distribution

Geographical locations were collected for each accepted and uncertain species. Mapping was performed at the resolution of TDWG level 3 regions [31]. In total, 229 TDWG countries were included; islands, Antarctica and glaciated countries were excluded (see the electronic supplementary material for details). A species was determined to be present if an identified specimen was recorded for a given TDWG country. Location records for accepted and uncertain species were downloaded from the Global Biodiversity Information Facility (GBIF) on 29 November 2012 and supplemented with data gathered from FAUNMAP [32] and the primary literature based on searches of species names and regional reviews as listed in the electronic supplementary material, table S1. Species range maps produced by experts were accepted as valid locations. GBIF data were cleaned to remove erroneous records based on the following criteria: species recorded on a continent with no dated site on the specified continent, records associated with animals in captivity, records within the open ocean and records with latitude and longitude of zero. For continentally extinct species, locations recorded from their extant range were removed. After locations in open ocean had been removed, locations for species occurring close to the coast were moved to the nearest TDWG country to account for lower sea levels during the Last Glacial. A presence/absence map, representing total cumulative range of each species across the study period (see the electronic supplementary material for further details), was plotted for each species (based on the electronic supplementary material, Data S1). The species distributions maps were typically discontinuous at least partly as a result of geographical gaps in the fossil record (electronic supplementary material, Data S1). These probably primarily reflect geographical bias in collection efforts as well as taphonomic bias, so we controlled for this by filling gaps in species ranges where: (i) the range was discontinuous (e.g. *Hippidion principale* and *Elephas namadicus*), (ii) there were holes within the range (e.g. Nevada, Colorado and Oklahoma for *Canis dirus*), and (iii) to fill peripheral TDWG countries on the edge of ranges (e.g. the Northern Provinces of Australia and the Guianas). For species-specific exceptions, see the electronic supplementary material. Where the gaps between records were extensive, gaps were filled on a path that best reflected environmental conditions in the regions where specimens were recovered. The primary analysis was performed including the filled records. To test the sensitivity of the results to our interpolations, the analysis was re-run using only the direct records.

### (c) Data analysis

The total number of extinct large mammals was calculated for each TDWG country (figure 1*b*). To account for naturally occurring species richness gradients and the uneven area of each TDWG country, extinction was expressed as a proportion of the total number of extinct and extant species (figure 1*a*). To remove the effects of human-driven extinctions and range contractions occurring in the last 1000 years, we estimated current extant large mammal richness for each TDWG country by accounting for the magnitude of human impact [33]. We initially created a general linear model (GLM), with a Quasi-family, logit link and variance of *μ*(1 − *μ*), with large mammal richness modelled as a function of continent, small mammal richness (International Union for Conservation of Nature range maps [34]) and human impact [33]. Six continents were included in our analysis; however, Asia was divided into two, tropical and temperate according to the TDWG classifications, and modelled individually. Human impact [33] was a significant predictor of large mammal diversity in North America (*p* ≤ 0.001), and temperate Asia (*p* ≤ 0.001), and approached significance in Europe (*p* = 0.062). For these regions, extant large mammal TDWG richness was estimated from this model using the lowest human impact recorded within the specific continent, and so within the predictive range of our models (electronic supplementary material, figure S11).

We focused on temperature and precipitation contrasts between the LGM, *ca* 21 000 years BP and the present-day interglacial climate, as this contrast represents the full glacial–interglacial amplitude of climate change during our study period. The chronology of extinctions, however, extends across the late Quaternary, including extinctions prior to the LGM [3]. To account for this, we also explored climate change between the LIG and the LGM as a supplementary analysis (electronic supplementary material, figure S10). Importantly, temperature and precipitation anomalies are highly correlated between the LIG–LGM and LGM–present time periods (0.97 and 0.82, respectively), indicating spatial consistency in the severity of climate change across the late Quaternary. Replacing the LGM–present climate contrasts with the LIG–LGM contrasts in a supplementary analysis provides even less support for the climate change hypothesis.

Climate change was measured using four variables, two measures of climate change velocity [21,22], the rate of displacement of the mean annual temperature and annual precipitation from the LGM to today, we measured the spatial gradient using a 2.5 arcminute resolution map, and two measures of climate change magnitude, LGM to present mean annual temperature anomaly and precipitation anomaly; these climate data were obtained from WorldClim at 2.5 arcminutes [35] (figure 1*d*,*e*; electronic supplementary material, figure S1). Temperature and precipitation anomaly were calculated by averaging records from the MIROC3 and CCSM models [36,37]. For each climate change variable, a standard measure was created by taking the square root of each data point and dividing by the maximum, creating scores between 0 and 1. For the measures of anomaly, absolute values were used as we are exploring the magnitude of change and not the direction. Although our analysis is at the TDWG country scale, species will probably respond to more local environments. Local-scale topography affects climate change velocities in particular and could vary considerably within region. To assess the degree of climate change velocity variation, velocity range was calculated for each TDWG country. The median velocity range per country was 16 m yr^−1^ for temperature and 44 m yr^−1^ for precipitation. This suggests that the velocity variation within each TDWG country was relatively small and unlikely to be problematic. To test for bias against climate explanatory variables measured on a continuous scale and for uncertainty in climate models, we used *k*-means clustering and a climate sensitivity test, respectively (see the electronic supplementary material).

### (d) Model selection

Climate change, hominin palaeobiogeography and hominin–climate combination models were constructed and compared to determine which predictor variables best describe the recorded regional extinction ratios. As velocity is calculated using climate change anomaly to avoid problems with collinearity, particularly in the temperature data, the velocity and anomaly of the same predictor variable were not included in the same climate or hominin–climate combination model. For the hominin models, we explored all combinations of hominin palaeobiogeography and the optimal description was found to be lumping the three Archaic regions together (based on an ordinal analysis of the five categories that revealed significant differences between *Homo*-origin and Archaic-early, and Archaic-peripheral and *H. sapiens*-only, but not between Archaic-early and Archaic-late and Archaic-peripheral), which we refer to as Archaic-combined. This combined model also performed better than including modern human arrival time [38] (based on Akaike information criterion (AIC)). We initially constructed GLMs using an arcsine square-root transformation of the response variable to account for the non-normal distribution of the proportion data. This transformation was deemed appropriate because the nature of these binomial data were not a factor of sampling effort and we wanted to give a 50% reduction of large mammal community within a TDWG country the same weight no matter if the reduction was from four to two species or 50–25 to avoid recording low extinction severity in regions with low total species diversity. We used AIC to select the best climate change, hominin palaeobiogeography and hominin–climate combined models. For comparison to the above approach, we also constructed a GLM using a Quasi-family, logit link and variance of *μ*(1 − *μ*), to account for the non-normal distribution of proportion data [39], finding the results to be consistent (see the electronic supplementary material). To assess the degree of spatial autocorrelation (SAC), we computed correlograms of GLM model residuals using the ‘ncf’ package in R [40], with distance classes of 500 km (electronic supplementary material, figure S3). A spatial autoregressive (SAR) model of the error type (SAR_err_ sensu [41]) was used to account for SAC in the multivariate hominin–climate model and compared to the GLM using the arcsine square-root transformation. The SAR models were implemented in the R package ‘spdep’ [42]. The spatial weights matrix was defined by connecting each TDWG country to its four closest neighbours. This SAR model removed residual SAC (electronic supplementary material, figure S3). All analyses were performed in R.v. 2.15.1 [43].

## 3. Results

Our extensive and taxonomically conservative literature review provided evidence of 177 globally or continentally extinct large mammal species (more than or equal to 10 kg; Africa 18, Asia 38, Australasia 26, Europe 19, North America 43 and South America 62; electronic supplementary material, table S1; figure 1*b*) for the period 132 000–1000 years BP. Of the 229 TDWG countries included in the analysis, the highest number of extinct species occurred in Texas (33 species), while Uruguay showed the highest proportion of extinct species (78%, figure 1*a*). Southern South America, southeast North America, western Europe and southern Australia emerged as extinction hotspots, with sub-Saharan Africa and southern Asia as notable cold spots (figure 1*a*). Thirty-one TDWG countries had no recorded extinctions.

Using climate change predictors only, a GLM including both temperature anomaly and precipitation velocity best described the extinction pattern, although with poor explanatory power (GLM: *F*_226_ = 29.790, *p* < 0.001; 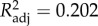). Human palaeobiogeography included as a categorical variable with three levels, *Homo*-origin, Archaic-combined and *H. sapiens*-only (figure 1*c*), had strong explanatory power (GLM: *F*_226_ = 201.240, *p* < 0.001; 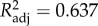). A full model was constructed from the predictors of the best climate and human models with interactions between human biogeography and the two climate predictors (GLM: *F*_220_ = 68.740, *p* < 0.001; 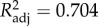; electronic supplementary material, figure S2 and table S2). The full model was significantly better than either the human or climate model (climate: *p* < 0.001; human: *p* < 0.001), but explanatory power was only weakly improved over the human-only model.

**Figure 2.**
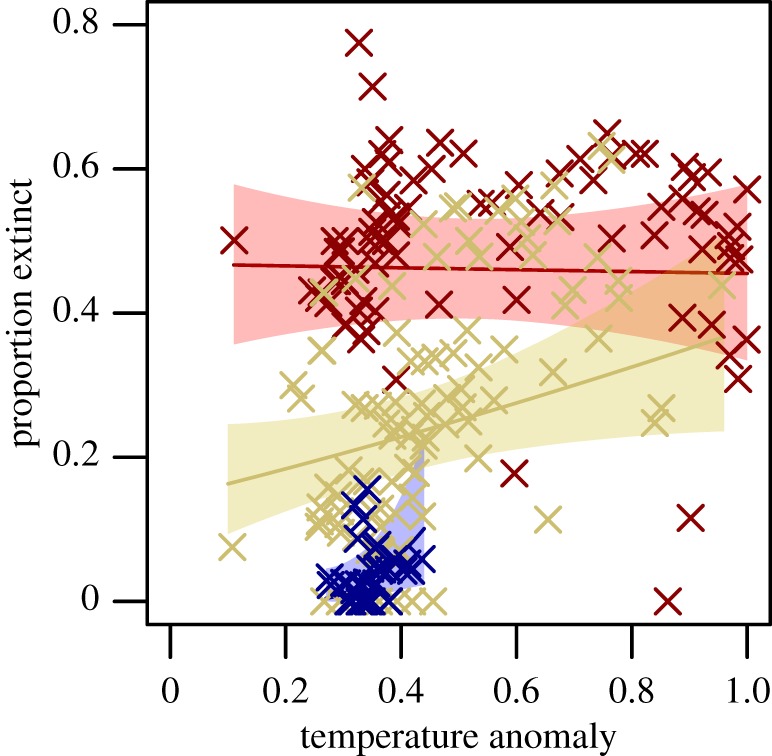
Proportion of extinct large mammals occurring in each TDWG country against mean annual temperature anomaly. Red crosses are countries occurring in the region of ‘*H. sapiens-*only’. Yellow crosses indicate countries occurring within the region ‘Archaic-combined’. Blue crosses indicate countries within the region ‘*Homo*-origin’. Lines of matching colours are predicted values based on a SAR model where hominin history and temperature anomaly with an interaction effect describe the proportion of extinct large mammals. Shaded areas represent 95% CIs. The statistical details are available in table 1.

**Figure 3.**
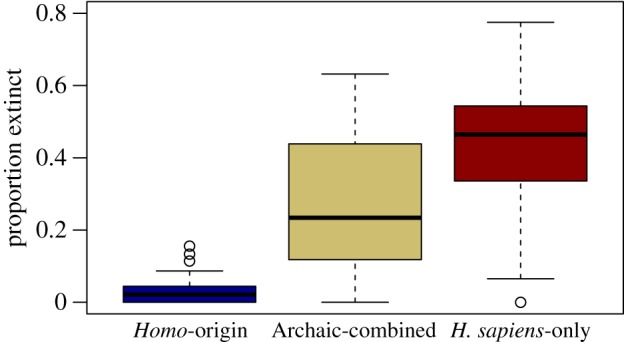
Box plot of proportion of large mammals lost per TWDG country in hominin palaeobiogeographic regions. Middle line and box represent the median and first to third quartiles, respectively, and whiskers extend to the furthest data point that is no more than 1.5 times the interquartile range.

Because of strong SAC in the residuals (electronic supplementary material, figure S3), the pairwise and multivariate analyses were all re-done accounting for SAC. A simultaneous autoregressive (SAR) model was used to account for SAC in the multivariate climate–hominin model. The best combined SAR model included hominin palaeobiogeography, temperature anomaly and an interaction between the two, while precipitation velocity was excluded (figure 2 and table 1). The model indicated significant effects of hominin palaeobiogeography, with intermediate extinctions in the Archaic-combined region and the most severe extinctions in the *H. sapiens*-only region (figure 3), temperature anomaly and their interaction. The interaction reflected the fact that the temperature anomaly effect was observed only in the Archaic-combined region (figure 2). These findings were robust to decisions related to the palaeontological information, distribution interpolation, extant species richness estimation and size threshold as well as to whether climate change was recorded as a categorical variable, was represented by the LIG–LGM shift rather than the LGM–present shift, and uncertainty in the climate models (electronic supplementary material, figures S4–S12, tables S3–S7 and see Sensitivity Analysis section).

**Table 1.** SAR model explaining global patterns in the proportion of extinct large mammals (more than or equal to 10 kg). (Est, regression coefficient; s.d., standard deviation; *Z*, test statistic; *p*, *p*-value; HS, *H. sapiens-*only region; AC, Archaic-combined region; TA, mean annual temperature anomaly between LGM and today, standardized to range between 0 and 1.)

	estimate	s.d.	*Z*	*p*
intercept	−0.353	0.263	−1.341	0.18
*H. sapiens-*only	1.107	0.274	4.037	<0.001
Archaic-combined	0.735	0.271	2.713	0.007
temperature anomaly	1.504	0.739	2.037	0.042
HS × TA	−1.516	0.749	−2.022	0.043
AC × TA	−1.225	0.752	−1.630	0.103
pseudo *R*^2^	0.635			
*p*	<0.001			
AIC	−272.57

This analysis illustrates that the late Quaternary megafauna extinctions were strongly linked to hominin palaeobiogeography and only weakly to glacial–interglacial climate change. The relationship between hominin palaeobiogeography and extinction magnitude is striking, with universally low extinctions in sub-Saharan Africa (maximum 13%), where hominins and the megafauna have long coexisted, but widespread exceptionally high extinctions in Australia and the Americas, where modern humans were the first hominin present. Only three TDWG countries in the *H. sapiens*-only region had extinction ratios below 30%, two of which are small, coastal states in North America with missing data as a likely cause of their low extinction values. The third is Japan, but the coding of hominin–palaeobiogeography for this country is potentially problematic because the country includes some endemic species that were never in contact with archaic humans, but many others with ranges that were, suggesting that the country could alternatively be coded as ‘Archaic-peripheral’. Eurasia falls between these extremes with a wide variety of extinction ratios, presenting a more complicated story. Globally, human palaeobiogeography alone accounts for 64% (GLM estimate) of the variation in extinction, while temperature anomaly, in combination with precipitation velocity in the best climate-only model, had much weaker explanatory power (20%) and only showed a relation to the proportion of extinct large mammals in the Archaic-combined region in the combined model.

## 4. Discussion

The global pattern of late Quaternary megafauna extinction presents a clear picture that extinction is closely tied to the geography of human evolution and expansion and at most weakly to the severity of climate change. The pattern of extinctions closely followed the hominin paleobiogeography hypothesis with increasing severity of extinction with reduced period of hominin–megafauna coevolution, notably with uniformly high extinction in areas where *H. sapiens* was the first hominin to arrive. By contrast, only in Eurasia was there a climate change signal.

High extinction despite a relatively stable climate is most striking in South America, forming a strong contrast to sub-Saharan Africa where extinction was minimal in spite of similar glacial–Holocene climate changes (figure 1*a*,*d*,*e* and the electronic supplementary material, figure S1). This relationship is particularly notable in southern South America where extinction ratios were among the highest recorded. Extinctions in the region have been linked to fluctuations between humid and dry periods and shifts in the availability of savannah and forest habitats. For instance, De Vivo & Carmignotto [44] associated a humid period in the mid-Holocene with loss of savannah and the demise of some megafauna. There is no indication, however, that these fluctuations were greater than in Africa or elsewhere [45]. Furthermore, dietary analyses of large South American herbivores such as *Eremotherium laurillardi*, *Stegomastodon waringi* and *Toxodon platensis* indicate that these extinct species were adaptable generalists, altering their diet between grazing and mixed feeding according to the habitat they occupied [46]. Additionally, other areas maintained grassland or forest throughout the whole period despite fluctuating climatic conditions [47]. The pampas region, for example, remained open throughout this time [47], but many megafauna species still disappeared, such as *Megatherium americanum*, which has been associated with human exploitation [48]. Extinctions in South America are poorly dated, but many have occurred after modern human arrival, approximately 15 000 years BP, and continued into the climatically relatively stable Holocene [19].

Most Australian extinctions occurred prior to the LGM, and so outside our LGM–present climate change predictor variable, just subsequent to the time humans are thought to have arrived (72–44 kyr BP) [3]. Australia experienced gradual drying from 400 ka that is proposed to have achieved a hydrological threshold in the last glacial; Wroe & Field [4] claim this drying drove a staggered extinction of many of the Pleistocene megafauna prior to the arrival of modern humans. Alternatively, it has been argued that modern humans caused the Australian megafauna extinctions either via fire-driven vegetation changes [49] or hunting [8]. Rule *et al*. [50] gave a detailed chronology for a dramatic decline in large mammal biomass in northeastern Australia during a period of climatic stability millennia after discernible drying events, after human arrival and prior to habitat change, supporting human-driven megafauna attrition as a driver of vegetation community change rather than the reverse (see also [17,51,52]). In agreement with this, we observe high extinction rates across Australia, but low to moderate temperature and precipitation anomalies and velocities (figure 1*a*,*d*,*e*). Additionally, LIG–LGM climate change measures indicated similarly moderate to low levels of relative temperature change across this period (electronic supplementary material, figure S10).

Our results also confirm earlier observations that extinctions could be severe even in relatively climatically stable regions in North America, where the vegetation changed little [53]. For instance, despite relatively little climate change and local survival of, for example, chapparel vegetation from LGM to today [54], California lost 21 megafauna species (53%, figure 1*a*,*b*) such as *Nothrotheriops shastense*, *Capromeryx minor* and *C. dirus* (see the electronic supplementary material, Data S1). In some cases, megafaunal species went extinct even though their preferred local diet has remained plentiful to the present-day, e.g. *Mammut americanum*, which fed on twigs of trees such as bald cypress (*Taxodium distichum*) in Florida [55]. *Euceratherium collinum* and *N. shastense* are further examples of species from western USA that went extinct despite the continued presence of favoured plant food sources in the region [56]. Grund *et al*. [57] highlights that the extinction pattern across North America is not consistent with climate as the main driver because species with small geographical ranges were not disproportionately affected. These megafauna patterns contrast with the global findings reported by Sandel *et al*. [21] that small-ranged amphibians, mammals (mostly small-sized) and birds are absent or scarce where Quaternary climate change has been severe. The period of extinction in North America occurred approximately 11 500–10 000 years BP, well within our period of measured climate change, and broadly occurred when climate change was severe, but also consistent with the arrival of modern humans [3].

Martin & Wright [6] were first to argue strongly for modern humans as the likely driver of megafauna extinctions worldwide, envisioning a swift overkill at the range front of expanding modern human populations. Since the emergence of that idea, other slower extinction models have been proposed involving anthropogenic fires and human-driven habitat change [49,50]. Although strong scepticism continues about humans as the extinction driver, primarily based on an argued scarcity of archaeological evidence of megafauna kills [4,16], others have shown that that kill sites are not rarer than expected [58,59] and that prehistoric human populations had the potential to exterminate megafauna populations [7]. Our analysis further supports humans as the key driver of extinction, but also has yielded the critical finding of little association between climate change severity and the proportion of extinct large mammals, suggesting that climate change was not a pervasive cause of this global-scale extinction event. Some earlier research has indicated that late Quaternary climate change affected megafauna species population dynamics and distributions [60], similar to the well-documented effects on smaller animals and plants [61]. Such effects, however, do not explain why most megafauna species did not go extinct where climate change was most severe or during previous glacial–interglacial cycles. In addition, although megafauna–hominin–climate interactions will have varied among species and regions, our analysis strongly supports modern human colonization of virgin territory as the more pervasive driver of extinction.

The temperature-related pattern in the Archaic-combined region is consistent with previous suggestions of climate as a major extinction driver there, especially in northern areas [3,60,62]. The relationship appears to be driven by high temperature anomaly and extinctions in northwest Eurasia and low-temperature anomaly and extinctions in southern Asia, but there are also widespread mismatches (figure 1*a*,*d*). However, the absence of a climate–extinction association from the *H. sapiens*-only region is inconsistent with extinctions resulting from synergism between climatic stress and hunting pressure because the association should rather have been strongest here. Instead, this effect appears consistent with human-driven extinctions with climatic constraints on archaic human distributions in northern parts of Eurasia [25], causing these regions to function as at least partial megafaunal refugia until modern human arrival [63]. At the same time, subtropical and tropical southern Asia were earlier, more permanently, and more densely colonized by archaic humans [24] and may thus be relatively similar to sub-Saharan Africa, with low late Quaternary extinctions because of either coevolution and/or pre-Late Pleistocene extinctions [28,64]. Such a scenario fits the extinction pattern for *Mammuthus primigenius* in Eurasia, with a range contraction towards high latitudes despite earlier temperate occurrences [65], and late survival in the high north beyond the range of modern humans, followed by sudden extinction with human arrival to these regions [60,63]. Eurasia experienced the most prolonged extinction during the Late Pleistocene with species lost before and after the LGM [3]. However, the pattern of regional climate change severity was similar between the LIG–LGM and LGM–present (electronic supplementary material, figure S10) so the relationship between climate change and extinction severity is expected to be consistent regardless of whether species were lost before or after the LGM.

Sub-Saharan Africa experienced mild late Quaternary extinctions, as would be expected with the length of megafauna–hominin co-occurrence in this region. It is also worth noting that there are some indications that a number of megafauna extinctions in sub-Saharan Africa occurred earlier, in the Early- to Mid-Pleistocene, possibly as a result of early hominin hunting or competition [66]. However, Africa also experienced minimal glacial–Holocene temperature change and fits the continental-scale pattern of low climate change and extinction severity that Nogués-Bravo *et al*. [20] described. At the same time, the area nevertheless experienced strong changes in precipitation, particularly in West Africa where extinction was exceptionally low (electronic supplementary material, figure S1; figure 1*a*). Given the already mentioned strong contrasts in extinctions between sub-Saharan Africa and South America despite similar climate changes, Africa conforms well to the extinction pattern expected from hominin palaeobiogeography.

Our analysis illustrates the value of taking a macroscale approach for understanding this global-scale extinction event, particularly highlighting the numerous discrepancies between the extent of climatic change and extinction severity at a regional scale. Furthermore, our findings indicate that the current low diversity in large mammals [2] in many continental areas is an anthropogenic phenomenon, not a natural one, with important implications for nature management [67].

## Supplementary Material

Supplementary Material

## Supplementary Material

Table S1

## Supplementary Material

Supplementary Data 1

## Supplementary Material

Supplementary Data 2

## Supplementary Material

R analysis script

## Data accessibility

The data reported in this paper are provided in the electronic supplementary material.
