# Supplementary material for: Global late Quaternary megafauna extinctions linked to humans, not climate change
Source: Proc Biol Sci. 2014 Jul 22;281(1787):20133254. doi: 10.1098/rspb.2013.3254 (PMC4071532; doi:10.1098/rspb.2013.3254)
Supplement: Supplementary Material [file rspb20133254supp1.docx]

**Supplementary Material**

**Species Distribution**

***TDWG countries***

‘TDWG countries’ generally correspond to countries, but the smallest nations are combined with larger neighbours and the largest countries, e.g. U.S.A., are sub-divided into states. They were created by the International Working Group on Taxonomic Databases for Plant Sciences as a geographic standard for species distributions that conforms closely to politically boundaries, for which spatial data for biodiversity specimens are often recorded, but of more similar geographic area than political countries, making them a suitable unit for the reported analysis. TDWG countries were excluded if they were in the Antarctic, an oceanic island or entirely covered by ice at the LGM. District of Columbia (U.S.A.) was also excluded on account of its very small size. A few additional TDWG countries in North America were also excluded despite containing minor ice-free areas where these areas were isolated from the rest of the ice-free landmass (Fig. 1a)

***Temporal resolution of species range maps***

Precise temporal data were unavailable for the majority of specimen locations mostly because these do not exist, and are not recorded in GBIF. Records from Faunmap and the primary literature were accepted if they were from a site associated or dated to our study period. Some sources did not distinguish between the Middle and Late Pleistocene and these records were generally included in our range estimates, except for *Homotherium latidens, Elasmotherium sibiricum* and *Soergelia minor* which have known range contractions between the Middle and Late Pleistocene [1, 2]. Hence, as a result of the limited temporal data and to reduce bias from under sampling, specimens from periods older than the study period are included within the analysis. However, because our study period encompasses both glacial and interglacial climates older records offer an indication of a species maximum potential extent during our study period.

***Species-specific exceptions to range mapping***

*Elephas iolensis* was not connected through interpolation as the literature indicated that its range was divided between Northern and Southern Africa in the Late Pleistocene [3]. *Cuon alpinus* was range filled in Europe so that it would be contiguous with its extant distribution in Asia. Denisovan humans were interpolated to meet the range of *Homo neanderthalensis* reflecting the hominin presence in this region [4].

**Sensitivity Analysis**

To compliment the original GLM analysis, where the response variable was arcsine square-root transformed, we constructed a GLM using the Quasi family, logit link and variance of mu(1 – mu), to account for the non-normal distribution of the proportion data. With this approach, the full model including hominin palaeobiogeography, temperature anomaly and precipitation velocity with interactions between hominin palaeobiogeography and both climate variables performed better than simplified alternatives. The pseudo *R^2^* for the full model was 0.715 which compared with 0.629 for the human only model and 0.201 for the climate model.

To account for uncertainty in the number of extinct species in our study period and to explore the effect of interpolating species ranges to create contiguous distributions we repeated the SAR analysis three times: 1) Excluding uncertain and interpolated species ranges, 2) Including uncertain and interpolated species ranges, and 3) Including uncertain but excluding interpolated ranges (Figure S4). The conclusions drawn in the main analysis are consistent with these supplementary analyses (Table S3; Figure S5). The model that best described these data included hominin palaeobiogeography, temperature anomaly and an interaction between the two for each of the three sensitivity tests. When the interpolated records were removed it is noticeable that there is an increased tendency for the proportional extinction to decline with increasing temperature anomaly within the *H. sapiens*-only region (Figure S5a & c), i.e., opposite of the predictions from the climate change hypothesis.

To test the sensitivity of our results to the extant species correction that we used to account for species extinctions and range contractions in the last 1000 years, we repeated the SAR analyses using proportions calculated from the observed extant large mammal richness (Figure S11b). Again the highest extinction was observed in the *H. sapiens*-only region (Figure S7), however, the model indicated non-significant interactions between temperature anomaly within the archaic-combined and *H. sapiens*-only regions and so maintaining the positive effect of temperature anomaly in both regions (Figure S7, Table S4). The divergence of this pattern from our main analysis is driven by the low large mammal diversity observed in south-eastern North America, a region heavily impacted by humans[5] and so receives a high correction factor, and is also a region where temperature anomaly is high (Figure 1c and Figure S11). This association between extinction and temperature anomaly appears to be related to more recent extinctions and range contractions from this particular region in south-eastern North America, unrelated to glacial-interglacial climate change.

Megafauna are often defined as species greater than or equal to 44 kg [6] and we repeated our analysis using this mass threshold. Of our accepted 177 species from the main analysis 154 species were identified as ≥44 kg. The global pattern of proportional extinct mammals was broadly similar to our main analysis (Figure S8). However, Australia recorded especially high extinction proportions as a result of very few extant mammals greater than 44 kg remaining (Figure S11). The model was consistent with the results of the main analysis with the most severe extinctions in the *H. sapiens*-only region and temperature anomaly effect in the archaic-combined region (Table S5, Figure S9).

To test the sensitivity of the results to the period of climatic change considered, we repeated the GLM and SAR analysis using climate change anomaly and velocity recorded between the Last Interglacial (LIG) and LGM (Figure S10). LIG climate data were downloaded from WorldClim at 30 arc seconds and aggregated to 2.5 arc minutes [7]. The GLM analysis indicated a model including hominin palaeobiogeography, temperature anomaly and an interaction between them best described the extinction pattern. However, the SAR modelling indicated that the hominin palaeobiogeography-only model best explained the extinction pattern (Table S6).

To test the importance of uncertainty in the climate models we performed a sensitivity test on the best climate-only and combined GLM models. Past climate data are available for two climate models, CCSM and MIROC. For each GLM, climate data were included by randomly selecting a value for each TDWG region between those reported for the CCSM and MIROC models. This process was repeated for 10,000 iterations. Of these 10,000, the model with the highest R^2^ was selected. Following this procedure the R^2^ for the combined model was increased to 0.714, an improvement of 0.01 from the original analyses. The climate only model the R^2^ was increased to 0.275, an improvement of 0.073. Using the best-fit climate data we repeated the SAR full model and recorded a pseudo-R^2^ of 0.707, an improvement of 0.07 from the original analysis. Despite modest improvements in model performance when selecting the best climate data to explain extinction, hominin paleobiogeography remained the strongest predictor and the conclusions remained the same indicating our results are robust to uncertainty in the modelled climate data.

Hominin palaeobiogeography was recorded as a categorical variable while climate was recorded as a continuous variable in the main analysis. To test the strength of all explanatory variables when recorded to the same precision we converted temperature anomaly and precipitation velocity, the climate variables that were included in the best original GLM model, and converted them to ordinal variables using k-means clustering with five levels (Figure S12). Mirroring the hominin palaeobiogeography approach a GLM was used to determine if there was a significant difference in the proportion of extinct large mammals between these ordinal levels. For temperature anomaly, level 1 (the region of lowest temperature anomaly) significantly differed from level 2, and 2 from 3, but 3 was not significantly different from 4, nor 4 from 5 (Table S7). There was no significant difference between levels for precipitation velocity (GLM: F = 0.1408, df = 224, *p* = 0.967; *R^2^_adj_* = -0.015). Replacing the continuous temperature anomaly variable with the clustered version (using 3 levels) did not improve the SAR model (based on AIC), leaving hominin palaeobiogeography as the only factor with significant explanatory power, supporting our original analysis.

In summary, all supplementary analyses support the conclusion that the magnitude of regional megafauna extinction is strongly linked to hominin palaeobiogeography and not climate change severity.

**References**

1. Reumer J.W., Rook L., Van Der Borg K., Post K., Mol D., De Vos J. 2003 Late Pleistocene survival of the saber-toothed cat Homotherium in Northwestern Europe. *Journal of Vertebrate Paleontology* **23**(1), 260-262.

2. Titov V.V., Tesakov A.S. 2010 Quaternary stratigraphy and paleontology of the southern Russia: connections between Europe, Africa and Asia. (p. 78. Rostov-on-Don, International Union for Quaternary Research, Section on European Quaternary Stratigraphy, Southern Scientific Centre, Russian Academy of Sciences Geological Institute).

3. Werdelin L., Sanders W.J. 2010 *Cenozoic Mammals of Africa*. Ewing, University of California Press.

4. Chauhan P.R. 2009 The lower Paleolithic of the Indian subcontinent. *Evolutionary Anthropology: Issues, News, and Reviews* **18**(2), 62-78.

5. Sanderson E.W., Jaiteh M., Levy M.A., Redford K.H., Wannebo A.V., Woolmer G. 2002 The human footprint and the last of the wild. *Bioscience* **52**(10), 891-904. (doi:10.1641/0006-3568(2002)052[0891:thfatl]2.0.co;2).

6. Barnosky A.D., Koch P.L., Feranec R.S., Wing S.L., Shabel A.B. 2004 Assessing the causes of Late Pleistocene extinctions on the continents. *Science* **306**(5693), 70-75.

7. Otto-Bliesner B.L., Marshall S.J., Overpeck J.T., Miller G.H., Hu A., members C.L.I.P. 2006 Simulating Arctic climate warmth and icefield retreat in the last interglaciation. *Science* **311**(5768), 1751-1753. (doi:10.1126/science.1120808).

**Supplementary Figures**


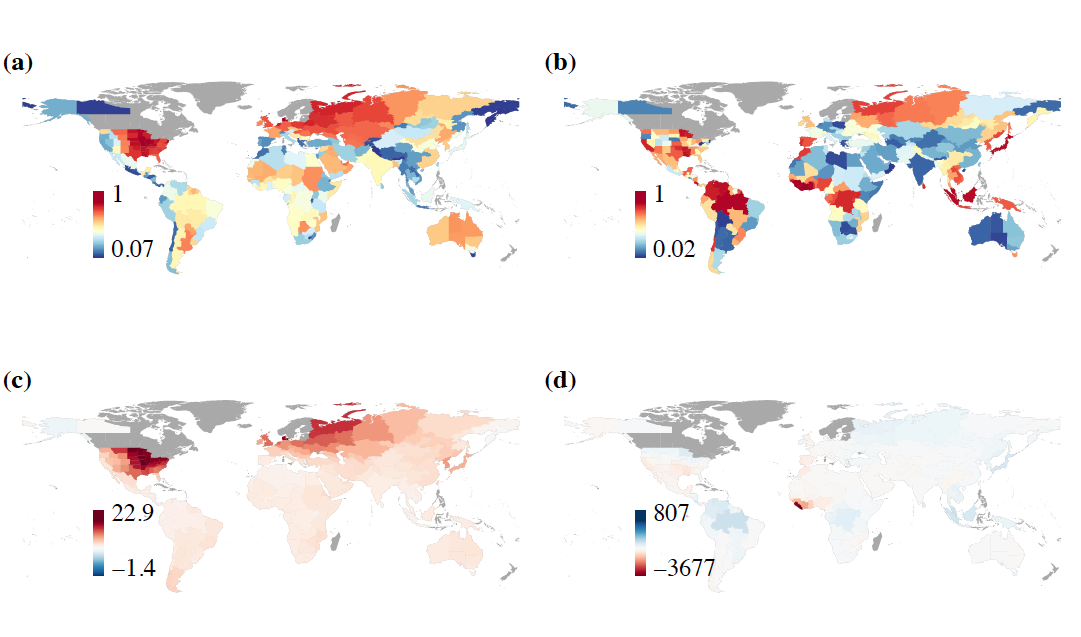


**Figure S1:** Precipitation anomaly and temperature anomaly and velocity in each TDWG country. a) Absolute mean annual temperature velocity between the Last Glacial Maximum (LGM) and today for each TDWG country, standardized to range between 0 and 1. b) Absolute mean anomaly in annual precipitation between the LGM and today for each TDWG country, standardized to range between 0 and 1. c) Raw mean anomaly in mean annual temperature (**°**C) between the LGM and today for each TDWG country. d) Raw mean anomaly in annual precipitation (mm per year) between the LGM and today for each TDWG country. Grey countries were excluded as they are either islands or were completely ice covered at the LGM and therefore have very poor Late Quaternary fossil records.

**Figure S2:** Ordinary least squares model predicting the proportion of extinct large mammals (≥10 kg) per TDWG country with arcsine square root transformation as a function of mean annual temperature anomaly between the Last Glacial Maximum and today, standardized to range between 0 and 1, and hominin palaeobiogeography. Red crosses and line indicate countries within the region first colonised modern humans ‘*H. sapiens*-only’. Yellow crosses and line indicate countries occurring within the region first colonised by ‘Archaic-combined’. Blue crosses and line are countries occurring in the region ‘*Homo*-origin’. Shaded areas represent standard error. The statistical details are available in table S2.

**Figure S3:** Correlogram comparing the best simultaneous autoregressive model, which includes hominin paleobiogeography (3 levels) and temperature anomaly (solid), with a general linear model using the same predictors (dashed).


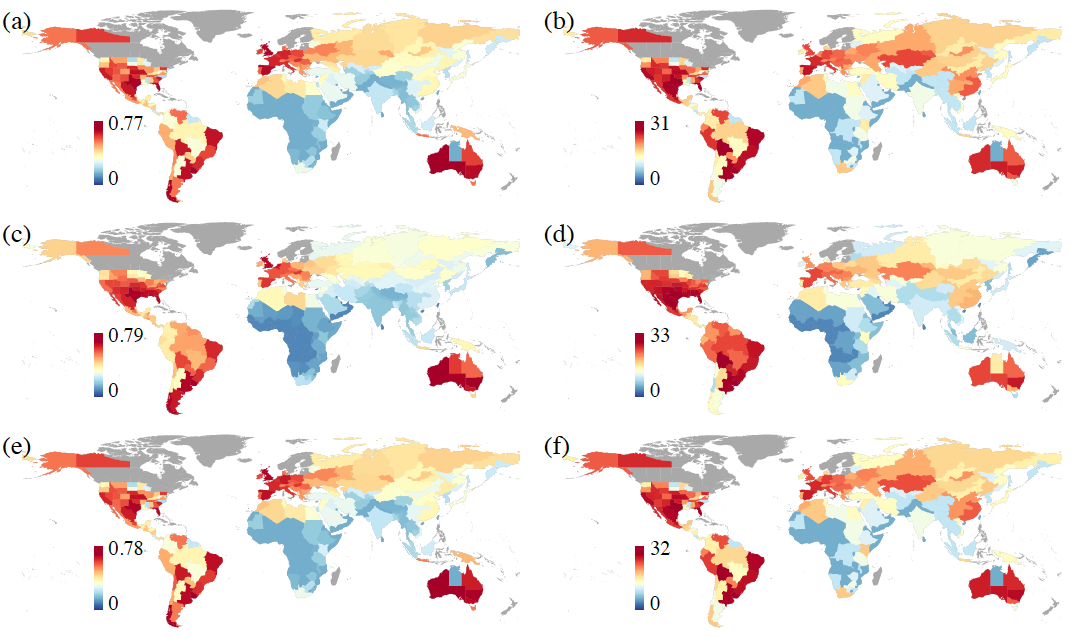


**Figure S4:** Number of and proportion of extinct large mammals per TDWG country varying the inclusion of uncertain species and interpolated records. a) The proportion of large mammal species (≥10 kg) extinct excluding uncertain species and interpolated ranges in each TDWG country during the last 132,000 years, only counting extinctions earlier than 1000 years BP. Grey countries were excluded from analyses as they are either islands or were completely ice covered at the Last Glacial Maximum and therefore have very poor Late Quaternary fossil records. b) The cumulative number of large mammal species excluding uncertain species and interpolated ranges occurring in each TDWG country. c) The proportion of large mammal species (≥10 kg) extinct including uncertain species and interpolated ranges in each TDWG country. d) The cumulative number of large mammal species including uncertain species and interpolated ranges occurring in each TDWG country. e) The proportion of large mammal species (≥10 kg) extinct including uncertain species and excluding interpolated ranges in each TDWG country. f) The cumulative number of large mammal species including uncertain species and excluding interpolated ranges occurring in each TDWG country.

**Figure S5:** Representations of a series of simultaneous autoregression models varying the inclusion of uncertain species and interpolated location records. Proportion of extinct large mammals (≥10 kg) occurring in each TDWG country predicted as a function of mean annual temperature anomaly between the last glacial maximum and today, standardized to range between 0 and 1 and hominin palaeobiogeography. Red crosses and line indicate countries within the region first colonised modern humans ‘*H. sapiens*-only’. Yellow crosses and line indicate countries occurring within the region first colonised by ‘Archaic-combined’. Blue crosses and line are countries occurring in the region ‘*Homo*-origin’. Shaded areas represent 95% confidence intervals. The statistical details are available for all models in table S3. a) The proportion of extinct species excludes uncertain species and interpolated locations. b) The proportion of extinct species includes uncertain species and interpolated locations. c) The proportion of extinct species includes uncertain species and excludes interpolated locations.

**
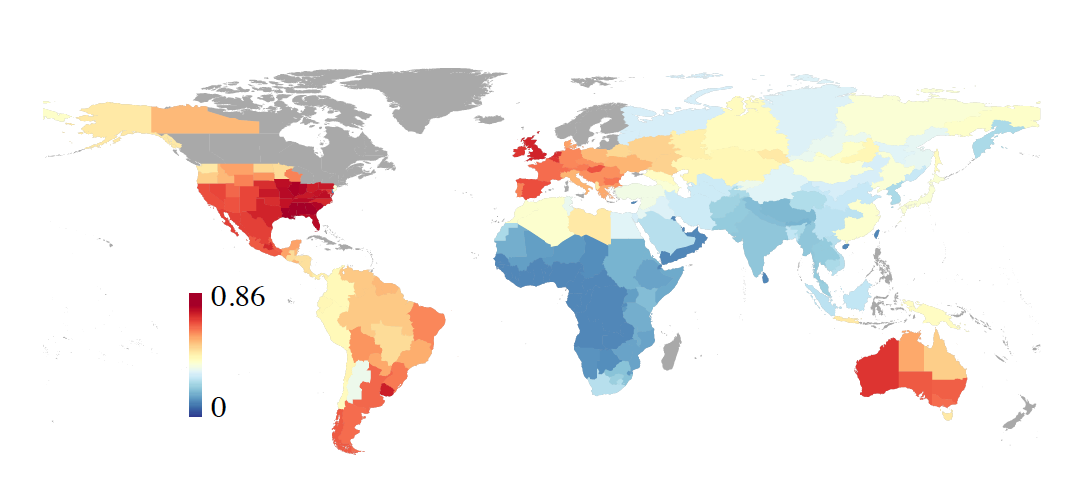
Figure S6:** The proportion of large mammal species (≥10 kg) extinct calculated using uncorrected extant large mammals (≥10 kg) in each TDWG country during the last 132,000 years, only counting extinctions earlier than 1000 years BP. Grey countries were excluded from analyses as they are either islands or were completed ice covered at the Last Glacial Maximum and therefore have very poor Late Quaternary fossil records.

**Figure S7:** Representation of a simultaneous autoregression model (SAR) explaining proportion of extinct large mammals (≥10 kg) calculated using uncorrected extant large mammal richness. Proportion of extinct large mammals occurring in each TDWG country predicted as a function of mean annual temperature anomaly between the last glacial maximum and today, standardized to range between 0 and 1 and hominin palaeobiogeography. Red crosses and line indicate countries within the region first colonised modern humans ‘*H. sapiens*-only’. Yellow crosses and line indicate countries occurring within the region first colonised by ‘Archaic-combined’. Blue crosses and line are countries occurring in the region ‘*Homo*-origin’. Lines of matching colours are predicted values based on a SAR where hominin history and temperature anomaly with an interaction effect describes proportion of extinct large mammals. Shaded areas represent 95% confidence intervals. The statistical details are available in table S4.


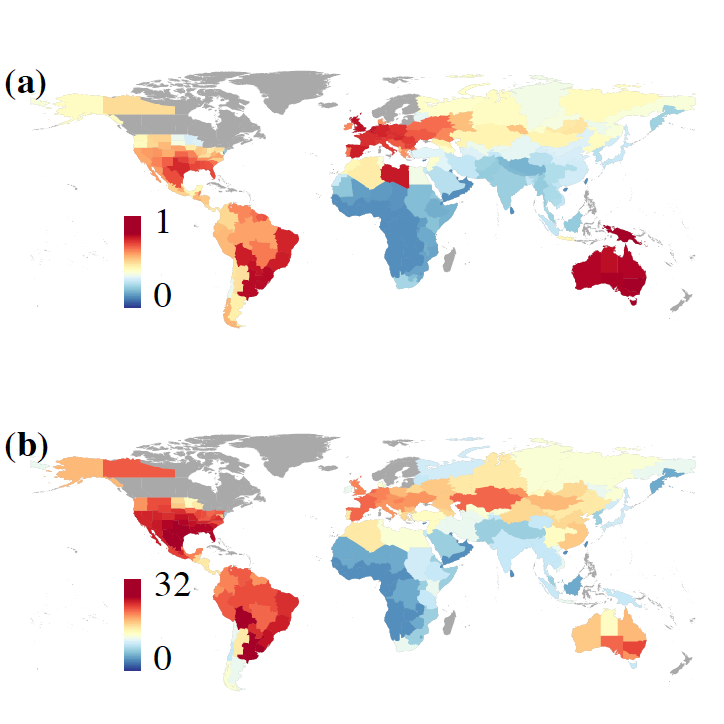


**Figure S8:** Number of and proportion of extinct megafauna (≥44 kg) per TDWG country. a) The proportion of megafauna species extinct excluding uncertain species but including interpolated ranges in each TDWG country during the last 132,000 years, only counting extinctions earlier than 1000 years BP. Grey countries were excluded from analyses as they are either islands or were completed ice covered at the Last Glacial Maximum and therefore have very poor Late Quaternary fossil records. b) The cumulative number of large mammal species excluding uncertain species and interpolated ranges occurring in each TDWG country during the last 132,000 years.

**Figure S9:** Representation of an simultaneous autoregression (SAR) model explaining the proportion of extinct megafauna (mammals ≥44 kg). Proportion of extinct megafauna occurring in each TDWG country predicted as a function of mean annual temperature anomaly between the last glacial maximum and today, standardized to range between 0 and 1 and hominin palaeobiogeography. Red crosses and line indicate countries within the region first colonised modern humans ‘*H. sapiens*-only’. Yellow crosses and line indicate countries occurring within the region first colonised by ‘Archaic-combined’. Blue crosses and line are countries occurring in the region ‘*Homo*-origin’. Lines of matching colours are predicted values based on a SAR where hominin history and temperature anomaly with an interaction effect describes proportion of extinct large mammals. Shaded areas represent 95% confidence intervals. The statistical details are available in table S5.

**
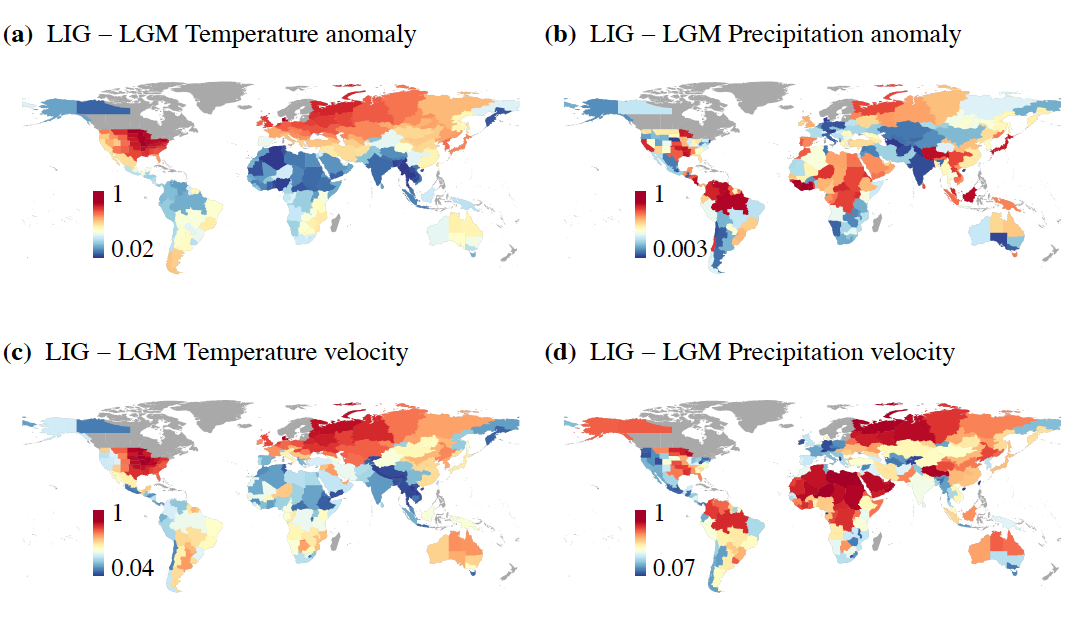
**

**Figure S10:** Precipitation and temperature anomalies and velocities in each TDWG country between the Last Interglacial (LIG) and Last Glacial Maximum (LGM). **a**) Mean anomaly in mean annual temperature between the LIG and the LGM for each TDWG country (climatic cold and warm extremes), standardized to range between 0 and 1. b) Mean anomaly in annual precipitation between the LIG and LGM and the present for each TDWG country, standardized to range between 0 and 1. c) Mean velocity in mean annual temperature between the LIG and the LGM for each TDWG country (climatic cold and warm extremes), standardized to range between 0 and 1. d) Mean velocity in annual precipitation between the LIG and LGM and the present for each TDWG country, standardized to range between 0 and 1.


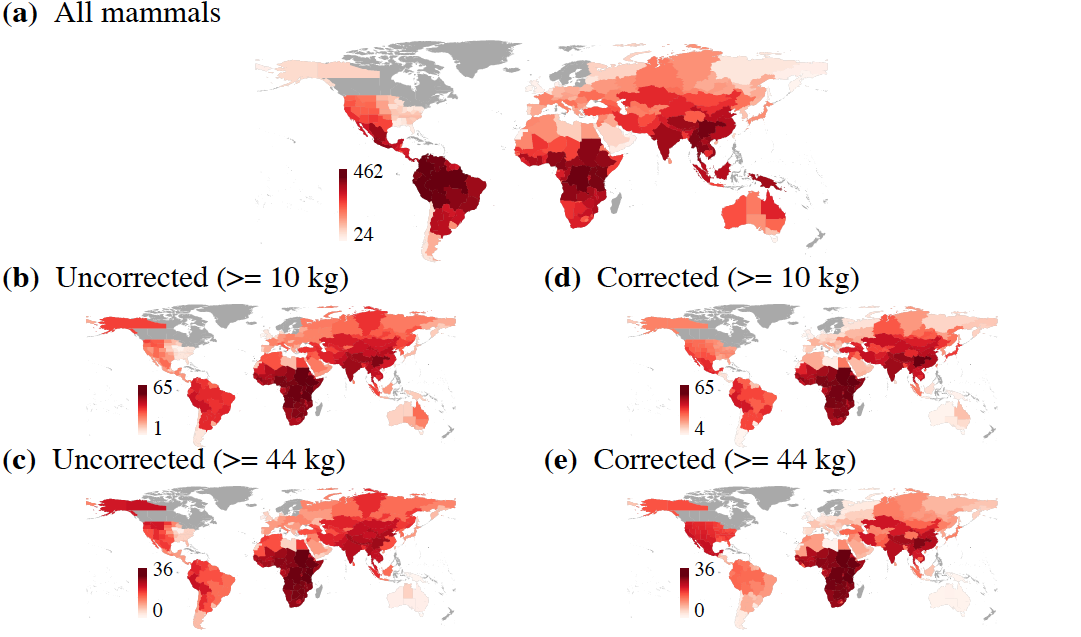


**Figure S11:** Extant species richness in each TDWG country. a) All extant mammals recorded from IUCN range maps. b) Recorded large mammal richness (≥10 kg) from IUCN range maps. c) Recorded large mammal richness (≥44 kg) from IUCN range maps. d) Estimated large mammal richness (≥10 kg) accounting for species extinctions and range contractions in the last 1000 years as a result of human impacts. e) Estimated megafauna mammal richness (≥44 kg) accounting for species extinctions and range contractions in the last 1000 years as a result of human impacts. Grey countries were excluded as they are either islands or were completely ice covered at the Last Glacial Maximum and therefore have very poor Late Quaternary fossil records.

**
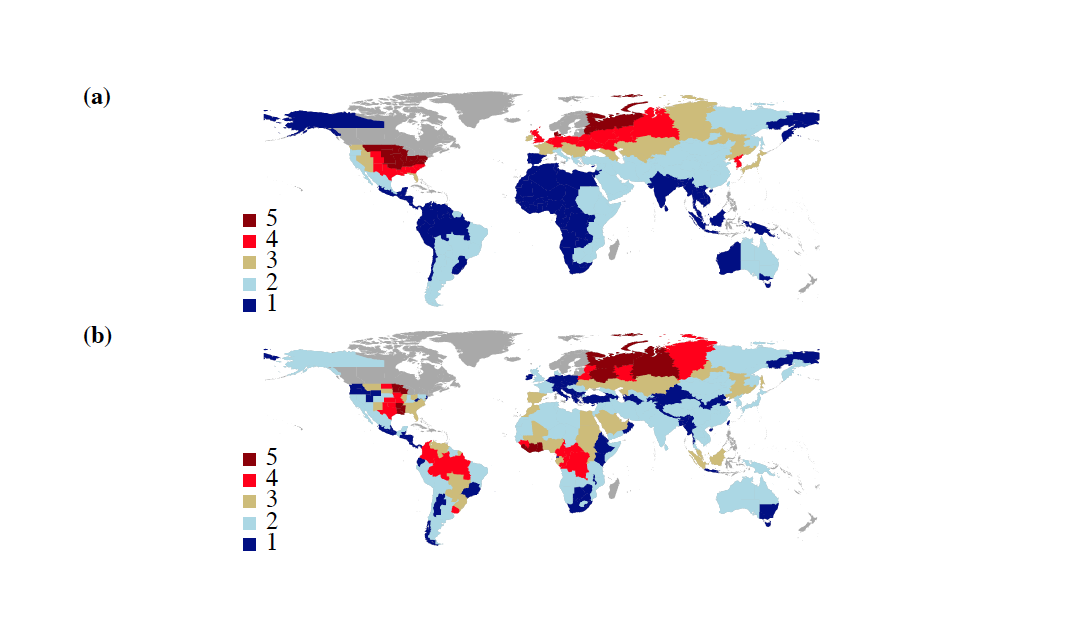
**

**Figure S12:** Clustered temperature anomaly and precipitation velocity variables. a) Temperature anomaly categorised by k-means clustering into five categories. b) Precipitation velocity categorised by k-means clustering into five categories. Categories 1 to 5 are ordered from lowest climatic change to highest. Grey countries were excluded as they are either islands or were completely ice covered at the Last Glacial Maximum and therefore have very poor Late Quaternary fossil records.

**Table S2:** General Linear Model (GLM) multiple regression and simultaneous autoregression (SAR) modelling results from combined climate and hominin models including precipitation velocity.

|  | **GLM** | | | | | **SAR** | | | |
| --- | --- | --- | --- | --- | --- | --- | --- | --- | --- |
|  | Est. | SD | T | *p* | Est. | | SD | Z | *p* |
| Intercept | 0.049 | 0.283 | 0.172 | 0.864 | -0.334 | | 0.271 | -1.233 | 0.218 |
| H. *sapiens*-only | 0.735 | 0.286 | 2.565 | 0.011 | 1.089 | | 0.283 | 3.846 | <0.001 |
| Archaic-combined | 0.178 | 0.288 | 0.617 | 0.538 | 0.694 | | 0.279 | 2.490 | 0.013 |
| Temperature anomaly | 0.487 | 0.773 | 0.630 | 0.529 | 1.543 | | 0.741 | 2.082 | 0.037 |
| Precipitation velocity | -0.271 | 0.123 | -2.204 | 0.029 | -0.068 | | 0.119 | -0.567 | 0.571 |
| HS x TA | -0.583 | 0.777 | -0.750 | 0.454 | -1.549 | | 0.752 | -2.059 | 0.039 |
| AC x TA | 0.258 | 0.780 | 0.331 | 0.741 | -1.333 | | 0.755 | -1.765 | 0.078 |
| HS x PV | 0.413 | 0.169 | 2.447 | 0.015 | 0.049 | | 0.162 | 0.301 | 0.764 |
| AC x PV | 0.114 | 0.170 | 0.669 | 0.504 | 0.250 | | 0.176 | 1.418 | 0.156 |
| F/Z | 68.740 | | | | 16.77 | | | | |
| pseudo R^2^ | 0.704 | | | | 0.674 | | | | |
| *p*-value | <0.001 | | | | <0.001 | | | | |

Model: Proportion of extinct megafauna = Hominin palaeobiogeography + Temperature anomaly + Precipitation velocity + Hominin palaeobiogeography × Temperature anomaly + Hominin palaeobiogeography × Precipitation velocity. Response variable was arcsine square root transformed. Est, regression coefficient; SD, standard deviation; F/Z, test statistic; *p*, *p*-value. HS, *H. sapiens*–only region. AC, archaic-combined region. TA, mean annual temperature anomaly between Last Glacial Maximum (LGM) and today, standardized between 0 and 1. PV, precipitation velocity between LGM and today, standardized between 0 and 1.

**Table S3:** Comparison between simultaneous autoregression models including or excluding uncertain species and location interpolations in explaining global patterns in the proportion of extinct large mammals (≥10 kg).

|  | **Exc. Uncertain, Exc. Interpolated** | | | | **Inc. Uncertain, Inc. Interpolated** | | | | **Inc. Uncertain, Exc. Interpolated** | | | |
| --- | --- | --- | --- | --- | --- | --- | --- | --- | --- | --- | --- | --- |
|  | Est. | SD | Z | *p* | Est. | SD | Z | *p* | Est. | SD | Z | *p* |
| Intercept | -0.339 | 0.340 | -0.997 | 0.319 | -0.387 | 0.266 | -1.454 | 0.146 | -0.352 | 0.342 | -1.027 | 0.305 |
| H. *sapiens*-only | 1.087 | 0.350 | 3.109 | 0.002 | 1.143 | 0.277 | 4.120 | <0.001 | 1.106 | 0.352 | 3.139 | 0.002 |
| Archaic-combined | 0.559 | 0.350 | 1.600 | 0.120 | 0.776 | 0.274 | 2.832 | 0.005 | 0.572 | 0.352 | 1.624 | 0.104 |
| Temperature anomaly | 1.244 | 0.971 | 1.280 | 0.200 | 1.626 | 0.747 | 2.178 | 0.029 | 1.283 | 0.979 | 1.311 | 0.190 |
| HS x TA | -1.440 | 0.981 | -1.468 | 0.142 | -1.639 | 0.758 | -2.163 | 0.030 | -1.485 | 0.989 | -1.502 | 0.133 |
| AC x TA | -0.767 | 0.987 | -0.777 | 0.437 | -1.353 | 0.760 | -1.780 | 0.075 | -0.805 | 0.994 | -0.809 | 0.418 |
| pseudo R^2^ | 0.559 | | | | 0.683 | | | | 0.558 | | | |
| *p*-value | <0.001 | | | | <0.001 | | | | <0.001 | | | |

Model: Proportion of extinct megafauna = Hominin palaeobiogeography + Temperature anomaly + Hominin palaeobiogeography × Temperature anomaly. Response variable was arcsine square root transformed. Est, regression coefficient. SD, standard deviation. Z, test statistic. HS, *H. sapiens*–only region. AC, archaic-combined region. TA, mean annual temperature anomaly between Last Glacial Maximum and today, standardized between 0 and 1.

**Table S4:** Simultaneous autoregression (SAR) model explaining global patterns in the proportion of extinct large mammals (≥10 kg) using observed as opposed to corrected extant richness.

|  | Est. | SD | Z | *p* |
| --- | --- | --- | --- | --- |
| Intercept | -0.368 | 0.306 | -1.206 | 0.228 |
| H. *sapiens*-only | 1.109 | 0.318 | 3.484 | <0.001 |
| Archaic-combined | 0.781 | 0.315 | 2.480 | 0.013 |
| Temperature anomaly | 1.588 | 0.859 | 1.849 | 0.065 |
| HS x TA | -1.369 | 0.871 | -1.572 | 0.116 |
| AC x TA | -1.293 | 0.874 | -1.479 | 0.139 |
| pseudo R^2^ | 0.698 | | | |
| *p*-value | <0.001 | | | |

Est, regression coefficient; SD, standard deviation; Z, test statistic; *p*, *p* value. HS, *H. sapiens*–only region. AC, archaic-combined region. TA, mean temperature anomaly between Last Glacial Maximum and today, standardized between 0 and 1.

**Table S5**: Simultaneous autoregressive (SAR) model explaining global patterns in the proportion of extinct large mammals (≥44 kg) using corrected extant richness.

|  |  | Est. | SD | Z | *p* |
| --- | --- | --- | --- | --- | --- |
| Intercept |  | -0.635 | 0.330 | -1.927 | 0.054 |
| *H. sapiens*-only |  | 1.603 | 0.345 | 4.646 | <0.001 |
| Archaic-combined |  | 1.191 | 0.339 | 3.513 | <0.001 |
| Temperature anomaly |  | 2.448 | 0.918 | 2.666 | 0.008 |
| HS × TA |  | -2.631 | 0.933 | -2.820 | 0.005 |
| AC × TA |  | -2.213 | 0.935 | -2.366 | 0.018 |
| pseudo R^2^ |  | 0.639 | | | |
| *p*-value |  | <0.001 | | | |

Est, regression coefficient. SD, standard deviation. Z, test statistic. *p*, *p* value. HS, *H. sapiens*–only region. AC, archaic-combined region. TA, mean temperature anomaly between Last Glacial Maximum and today, standardized between 0 and 1.

**Table S6**: Simultaneous autoregression (SAR) model explaining global patterns in the proportion of extinct large mammals (≥10 kg) using hominin palaeobiogeography and climate change data between the Last Interglacial (LIG) and the Last Glacial Maximum (LGM).

|  |  | Est. | SD | Z | *p* |
| --- | --- | --- | --- | --- | --- |
| Intercept |  | 0.198 | 0.060 | 3.318 | 0.001 |
| *H. sapiens*-only |  | 0.542 | 0.074 | 7.321 | <0.001 |
| Archaic-combined |  | 0.305 | 0.064 | 4.749 | <0.001 |
|  |  |  |  |  |  |
| pseudo R^2^ |  | 0.663 | | | |
| *p*-value |  | <0.001 | | | |

The table displays the final results of the best model, which does not include any climate variables. Est, regression coefficient. SD, standard deviation. Z, test statistic. *p*, *p* value. HS, *H. sapiens*–only region.

**Table S7**: General Linear Model using forward difference coding to determine if sequential ordinal categories of clustered temperature anomaly significantly differ.

|  |  | Est. | SD | Z | *p* |
| --- | --- | --- | --- | --- | --- |
| Intercept |  | 0.617 | 0.021 | 30.003 | <0.001 |
| Temp Anomaly Level 1 |  | -0.092 | 0.043 | -2.129 | 0.034 |
| Temp Anomaly Level 2 |  | -0.230 | 0.061 | -3.458 | 0.001 |
| Temp Anomaly Level 3 |  | -0.093 | 0.077 | -1.218 | 0.225 |
| Temp Anomaly Level 4 |  | 0.087 | 0.078 | 1.117 | 0.265 |
| pseudo R^2^ |  | 0.214 | | | |
| *p*-value |  | <0.001 | | | |

Est, regression coefficient. SD, standard deviation. Z, test statistic. *p*, *p* value.
