# Supplementary material for: Global late Quaternary megafauna extinctions linked to humans, not climate change
Source: Proc Biol Sci. 2014 Jul 22;281(1787):20133254. doi: 10.1098/rspb.2013.3254 (PMC4071532; doi:10.1098/rspb.2013.3254)
Supplement: Table S1 [file rspb20133254supp2.docx]

**Table S1:** Species list of accepted and uncertain globally and continentally extinct large terrestrial mammals (≥10 kg) occurring with the analysed TDWG country and occurring between 132,000 and 1,000 years BP. Date and location references are provided for each species. * *Bison bonasus* is a grouping of late quaternary specimens from the genus *Bison* occurring in Asia following the Eurasian bison IUCN taxonomy and taking a conservative approach to the number of bison species in the region.

| Species | Status | Data location sources | Dated reference | Mass reference |
| --- | --- | --- | --- | --- |
| *Agalmaceros blicki* | Accepted | [1, 2] | [2] | [3] |
| *Alces scotti* | Accepted | [4, 5] | [5] | [3] |
| *Antidorcas australis* | Accepted | [6] | [7] | [3] |
| *Antidorcas bondi* | Accepted | [8] | [7] | [3] |
| *Antifer ultra* | Accepted | [9, 10] | [10] | [3] |
| *Arctodus simus* | Accepted | [4, 5, 11] | [12] | [3] |
| *Arctotherium tarijense* | Accepted | [12] | [12] | [13] |
| *Arctotherium wingei* | Accepted | [12] | [12, 14] | [15] |
| *Bison bonasus** | Accepted | [16-18] | [19] | [3] |
| *Bootherium bombifrons* | Accepted | [4, 5, 20] | [21] | [3] |
| *Borungaboodie hatcheri* | Accepted | [22] | [23] | [24] |
| *Bos primigenius* | Accepted | [25] | [26] | [27] |
| *Bubalus palaeokerabau* | Accepted | [28] | [29] | [28] |
| *Caipora bambuiorum* | Accepted | [30] | [30] | [3] |
| *Camelops hesternus* | Accepted | [4, 5, 11, 20, 31] | [31] | [3] |
| *Camelus thomasi* | Accepted | [32] | [32-34] | [3] |
| *Canis dirus* | Accepted | [5, 11, 35] | [36] | [3] |
| Caprine sp. | Accepted | [37] | [37] | Estimated from Caprini |
| *Capromeryx minor* | Accepted | [4, 5, 38] | [5, 39] | [3] |
| *Castoroides ohioensis* | Accepted | [4, 5, 40] | [5] | [3] |
| *Catagonus stenocephalus* | Accepted | [41, 42] | [41] | [3] |
| *Catonyx cuvieri* | Accepted | [43-45] | [43] | [46] |
| *Coelodonta antiquitatis* | Accepted | [17, 18, 47] | [18] | [3] |
| *Crocuta crocuta* | Accepted | [18, 47-52] | [49] | [3] |
| *Cuon alpinus* | Accepted | [4, 47, 53] | [54] | [3] |
| *Cuvieronius hyodon* | Accepted | [4, 47, 55-57] | [58] | [3] |
| *Damaliscus hypsodon* | Accepted | [59] | [60] | [60] |
| *Damaliscus niro* | Accepted | [37] | [37] | Estimated from *D. hypsodon* |
| *Dasypus bellus* | Accepted | [4, 5] | [61] | [3] |
| *denisovan humans* | Accepted | [62, 63] | [62] | [64] |
| *Diabolotherium nordenskioldi* | Accepted | [65, 66] | [65] | [66] |
| *Diprotodon optatum* | Accepted | [22] | [22] | [3] |
| *Doedicurus clavicaudatus* | Accepted | [67] | [67] | [3] |
| *Dusicyon avus* | Accepted |  | [68] | [3] |
| *Elasmotherium sibiricum* | Accepted | [69] | [70] | [71] |
| *Elephas antiquus* | Accepted | [72-76] | [74] | [77] |
| *Elephas iolensis* | Accepted | [78, 79] | [80] | [3] |
| *Elephas namadicus* | Accepted | [48, 72, 81, 82] | [83] | [77] |
| *Elephas naumanni* | Accepted | [72, 84-86] | [87] | [77] |
| *Equus ferus* | Accepted | [5, 16, 88] | [5] | [3] |
| *Equus ovodovi* | Accepted | [69] | [89] | [27] |
| *Equus semiplicatus (stiltlegged horse)* | Accepted | [90] | [90] | [3] |
| *Eremotherium laurillardi* | Accepted | [5, 11, 40, 43, 55, 91-93] | [93] | [3] |
| *Euceratherium collinum* | Accepted | [4] | [21] | [3] |
| *Eutatus seguini* | Accepted |  | [94] | [3] |
| *Gazella atlantica* | Accepted |  | [39] | [3] |
| *Gazella tingitana* | Accepted | [37] | [37] | Estimated from *G. atlantica* |
| *Glossotherium robustum* | Accepted | [9] | [95] | [3] |
| *Glyptodon clavipes* | Accepted | [9, 43, 44, 91, 96] | [9] | [3] |
| *Glyptodon reticulatus* | Accepted | [44] | [97] | [3] |
| *Glyptotherium cylindricum* | Accepted | [11, 98] | [99] | [100] |
| *Glyptotherium floridanum* | Accepted | [5] | [5] | [3] |
| *Hemiauchenia macrocephala* | Accepted | [5] | [5] | [3] |
| *Hemiauchenia paradoxa* | Accepted | [9, 101] | [9] | [3] |
| *Hemitragus cedrensis* | Accepted | [102, 103] | [102] | [104] |
| *Hexaprotodon sivalensis* | Accepted | [29, 48] | [29] | [105] |
| *Hippidion devillei* | Accepted | [89, 93, 106] | [89] | [3] |
| *Hippidion principale* | Accepted | [9, 43, 89, 106] | [89] | [3] |
| *Hippopotamus amphibius* | Accepted | [107] | [108] | [3] |
| *Holmesina occidentalis* | Accepted | [109] | [109] | [3] |
| *Holmesina paulacoutoi* | Accepted | [109] | [109] | [3] |
| *Holmesina septentrionalis* | Accepted | [5] | [109] | [3] |
| *Homo erectus* | Accepted | [110] | [110] | [64] |
| *Homo neanderthalensis* | Accepted | [62] | [62] | [64] |
| *Homotherium latidens* | Accepted | [47, 111] | [111] | [3] |
| *Homotherium serum* | Accepted | [4, 5] | [5] | [3] |
| *Hoplophorus euphractus* | Accepted | [43] | [43] | [3] |
| *Hystrix kiangsenensis* | Accepted | [112] | [113] | [112] |
| *Hystrix refossa* | Accepted | [112] | [114] | [112] |
| *Kolpochoerus sp.* | Accepted | [37] | [37] | Estimated from *K. majus* |
| *Lestodon armatus* | Accepted | [9, 44] | [9] | [3] |
| *Macaca sylvanus* | Accepted | [115] | [116] | [3] |
| *Macrauchenia patachonica* | Accepted | [9, 117, 118] | [9] | [3] |
| *Macropus ferragus* | Accepted | [22] | [22] | [3] |
| *Mammut americanum* | Accepted | [4, 5, 57] | [5] | [3] |
| *Mammuthus columbi* | Accepted | [4, 5, 57] | [5] | [119] |
| *Mammuthus primigenius* | Accepted | [5, 17, 18, 47, 120-125] | [5] | [3] |
| *Manis paleojavanica* | Accepted | [126] | [127] | [128] |
| *Maokopia ronaldi* | Accepted | [22] | [22] | [3] |
| *Megalibgwilia ramsayi* | Accepted | [22] | [129] | [3] |
| *Megaloceros algericus* | Accepted | [130-134] | [132] | [3] |
| *Megaloceros giganteus* | Accepted | [18, 47, 102, 135] | [18] | [3] |
| *Megalonyx jeffersonii* | Accepted | [4, 5, 11, 40] | [5] | [3] |
| *Megalotragus priscus* | Accepted | [6] | [21, 59] | [3] |
| *Megalovis guangxiensis* | Accepted |  | [136] | Estimated from *Soergelia minor* |
| *Megatherium americanum* | Accepted | [9, 117] | [67] | [3] |
| *Megatherium tarijense* | Accepted | [137] | [137] | [138] |
| *Metasthenurus newtonae* | Accepted | [139] | [129] | [24] |
| *Metridiochoerus compactus* | Accepted | [140] | [140] | [3] |
| *Miracinonyx trumani* | Accepted | [4, 5, 40] | [5] | [3] |
| *Mixotoxodon larensis* | Accepted | [11, 55, 141] | [14] | [3] |
| *Morenelaphus brachyceros* | Accepted | [9] | [9] | [3] |
| *Morenelaphus lujanensis* | Accepted | [9] | [9] | [3] |
| *Mylodon darwinii* | Accepted |  | [68] | [142] |
| *Mylohyus nasutus* | Accepted | [4, 5] | [5] | [3] |
| *Navahoceros fricki* | Accepted | [4, 5, 11] | [5, 143] | [3] |
| *Neochoerus aesopi* | Accepted | [5, 9, 11, 144] | [5] | [3] |
| *Neolicaphrium recens* | Accepted | [9, 118] | [9] | [145] |
| *Neuryurus trabeculatus* | Accepted | [146] | [9, 146] | [147] |
| *Nothrotheriops shastense* | Accepted | [4, 11, 148] | [149] | [3] |
| *Nothrotherium maquinense* | Accepted | [30, 43, 44] | [30] | [3] |
| *Oreamnos harringtoni* | Accepted | [4, 150, 151] | [150] | [3] |
| *Ovibos moschatus* | Accepted | [4, 49, 152] | [153] | [3] |
| *Palaeolama major* | Accepted | [43, 101] | [154] | [3] |
| *Palaeolama mirifica* | Accepted | [5] | [5] | [3] |
| *Palaeolama weddelli* | Accepted | [101] | [155] | [3] |
| *Palorchestes azael* | Accepted | [22] | [22] | [3] |
| *Pampatherium humboldti* | Accepted | [9, 43, 109] | [109] | [3] |
| *Pampatherium typum* | Accepted | [109] | [109] | [3] |
| *Panochthus tuberculatus* | Accepted | [9] | [9] | [3] |
| *Panthera atrox* | Accepted | [4, 5, 11, 156] | [5] | [3] |
| *Panthera leo* | Accepted | [157] | [157] | [3] |
| *Panthera pardus* | Accepted | [18, 157] | [153] | [3] |
| *Panthera spelaea* | Accepted | [4, 18, 152, 156, 158] | [153] | [3] |
| *Paraceros fragilis* | Accepted | [1, 9] | [9] | [3] |
| *Paramylodon harlani* | Accepted | [11, 40, 159] | [95] | [3] |
| *Pelorovis antiquus* | Accepted | [6, 160, 161] | [21] | [3] |
| *Phascolomys medius* | Accepted | [162] | [162] | [3] |
| *Phascolonus gigas* | Accepted | [22] | [22] | [3] |
| *Platygonus compressus* | Accepted | [4, 5, 11, 40] | [5] | [3] |
| *Procoptodon browneorum* | Accepted | [139] | [22] | [24] |
| *Procoptodon gilli* | Accepted | [139] | [22] | [24] |
| *Procoptodon goliah* | Accepted | [22, 139] | [22] | [3] |
| *Propleopus oscillans* | Accepted | [22, 162] | [162] | [3] |
| *Propraopus punctatus* | Accepted |  | [61] | [61] |
| *Propraopus sulcatus* | Accepted | [61] | [61] | [61] |
| *Protemnodon anak* | Accepted | [22, 129, 163] | [129] | [3] |
| *Protemnodon brehus* | Accepted | [22, 163] | [22] | [3] |
| *Protemnodon hopei* | Accepted | [22] | [22] | [24] |
| *Protemnodon nombe* | Accepted | [24] | [164] | [24] |
| *Protemnodon roechus* | Accepted | [22] | [22] | [3] |
| *Protemnodon tumbuna* | Accepted | [24] | [164] | [3] |
| *Protocyon troglodytes* | Accepted | [30, 43] | [36] | [3] |
| *Protopithecus brasiliensis* | Accepted | [30, 43] | [30] | [27] |
| *Rusingoryx atopocranion* | Accepted | [59] | [59] | [59] |
| *Saiga tatarica* | Accepted | [5, 47, 49] | [165] | [3] |
| *Sangamona fugitiva* | Accepted | [5, 166] | [166] | [3] |
| *Scelidodon chiliensis* | Accepted | [67] | [67] | [3] |
| *Scelidotherium leptocephalum* | Accepted |  | [167] | [3] |
| *Sclerocalyptus ornatus* | Accepted |  | [168] | [3] |
| *Simosthenurus occidentalis* | Accepted | [139] | [22] | [3] |
| *Sinomegaceros ordosianus* | Accepted | [17, 133] | [169] | Esimated from *S. yabei* |
| *Sinomegaceros yabei* | Accepted | [17, 85] | [170] | [119] |
| *Smilodon fatalis* | Accepted | [4, 5, 11] | [5] | [3] |
| *Smilodon populator* | Accepted | [9, 30, 43, 44, 171, 172] | [67] | [3] |
| *Soergelia minor* | Accepted | [69] | [69] | [3] |
| *Spirocerus kiakhtensis* | Accepted | [173-178] | [173] | [3] |
| *Stegodon orientalis* | Accepted | [48] | [179] | [119] |
| *Stegodon trigonocephalus* | Accepted | [29, 48] | [29] | [180] |
| *Stegomastodon platensis* | Accepted | [56, 117, 181] | [58] | [3] |
| *Stegomastodon waringi* | Accepted | [56, 91] | [58] | [3] |
| *Stephanorhinus hemitoechus* | Accepted | [51, 72] | [153] | [3] |
| *Stephanorhinus kirchbergensis* | Accepted | [18, 72, 182, 183] | [153] | [3] |
| *Sthenurus andersoni* | Accepted | [22, 139] | [22] | [3] |
| *Sthenurus atlas* | Accepted | [22, 139] | [22] | [3] |
| *Sthenurus stirlingi* | Accepted | [22, 139] | [22] | [3] |
| *Sthenurus tindalei* | Accepted | [22, 139] | [22] | [3] |
| *Stockoceros conklingi* | Accepted | [5, 11] | [5] | [3] |
| *Sus brachygnathus* | Accepted | [29] | [29] | [184] |
| *Tapirus augustus* | Accepted | [48] | [39] | [119] |
| *Tapirus copei* | Accepted | [5, 11] | [185] | [3] |
| *Tapirus rondoniensis* | Accepted | [91] | [91] | [91] |
| *Tapirus veroensis* | Accepted | [5, 186, 187] | [5] | [3] |
| *Tetrameryx shuleri* | Accepted | [5, 11] | [5] | [3] |
| *Theriodictis tarijensis* | Accepted |  | [36] | [3] |
| *Thylacoleo carnifex* | Accepted | [22] | [22] | [3] |
| *Toxodon platensis* | Accepted | [9, 43, 44, 117, 141] | [97] | [3] |
| *Tremarctos floridanus* | Accepted | [5, 55] | [12] | [3] |
| *Trigonodops lopesi* | Accepted | [43, 91] | [91] | Estimated from *Toxodon platensis* |
| *Troposodon minor* | Accepted | [163] | [188] | [3] |
| *Ursus spelaeus* | Accepted | [18, 47, 189] | [189] | [190] |
| *Valgipes deformis* | Accepted | [46] | [46] | [3] |
| *Vombatus hacketti* | Accepted | [22] | [22] | [3] |
| *Wallabia kitcheneri* | Accepted | [22] | [22] | [24] |
| *Xenorhinotherium bahiense* | Accepted | [118, 191] | [99] | [192] |
| *Zaglossus hacketti* | Accepted | [22] | [22] | [3] |
| *Zygomaturus trilobus* | Accepted | [22] | [22] | [3] |
| *Aepyceros sp.* | Uncertain |  |  |  |
| *Bubalus murrensis* | Uncertain |  |  |  |
| *Catagonus bonaerensis* | Uncertain |  |  |  |
| *Eulamaops paralellus* | Uncertain |  |  |  |
| *Holmesina majus* | Uncertain |  |  |  |
| *Kolopsis watutense* | Uncertain |  |  |  |
| *Lestodon trigonidens* | Uncertain |  |  |  |
| *Macaca robustus* | Uncertain |  |  |  |
| *Megatherium medinae* | Uncertain |  |  |  |
| *Megatherium urbinai* | Uncertain |  |  |  |
| *Mylodopsis ibseni* | Uncertain |  |  |  |
| *Nycterutes vinetorum* | Uncertain |  |  |  |
| *Orcteropus crassidens* | Uncertain |  |  |  |
| *Pachyarmatherium brasiliense* | Uncertain |  |  |  |
| *Pampatherium mexicanum* | Uncertain |  |  |  |
| *Panochthus frenzelianus* | Uncertain |  |  |  |
| *Panochthus morenoi* | Uncertain |  |  |  |
| *Parapanochthus jaguaribensis* | Uncertain |  |  |  |
| *Plaxhaplous canaliculatus* | Uncertain |  |  |  |
| *Procoptodon oreas* | Uncertain |  |  |  |
| *Procoptodon pusio* | Uncertain |  |  |  |
| *Procoptodon williamsi* | Uncertain |  |  |  |
| *Simosthenurus pales* | Uncertain |  |  |  |
| *Sinomegaceros baotouensis* | Uncertain |  |  |  |
| *Sthenurus murrayi* | Uncertain |  |  |  |
| *Tapirus cristatellus* | Uncertain |  |  |  |
| *Tapirus mesopotamicus* | Uncertain |  |  |  |

**Table S1:** Species list of accepted and uncertain globally and continentally extinct large terrestrial mammals (≥10 kg) occurring with the analysed TDWG country and occurring between 132,000 and 1,000 years BP. Date and location references are provided for each species. * *Bison bonasus* is a grouping of late quaternary specimens from the genus *Bison* occurring in Asia following the Eurasian bison IUCN taxonomy and taking a conservative approach to the number of bison species in the region.

**References**

1. Merino M.L., Rossi R.V. 2010 Origin, systematics, and morphological radiation. In *Neotropical cervidology: Biology and Medicine of Latin American Deer* (eds. Duarte M.B., González S.V.), pp. 2-11. Jaboticabal, Brazil and Gland, Switzerland.

2. Tomiati C., Abbazzi L. 2002 Deer fauna from Pleistocene and Holocene localities of Ecuador (South America). *Geobios-Lyon* **35**(5), 631-645. (doi:10.1016/s0016-6995(02)00075-x).

3. Smith F.A., Lyons S.K., Ernest S.K.M., Jones K.E., Kaufman D.M., Dayan T., Marquet P.A., Brown J.H., Haskell J.P. 2003 Body mass of late Quaternary mammals. *Ecology* **84**(12), 3403-3403. (doi:10.1890/02-9003).

4. Kurtén B., Anderson E. 1980 *Pleistocene Mammals of North America*. New York, Columbia University Press.

5. Faunmap working group. 1994 FAUNMAP: a database documenting late Quaternary distributions of mammal species in the United States. *Illinois State Museum Scientific Papers* **25**(1-2), 1-690.

6. Faith J.T. 2011 Ungulate community richness, grazer extinctions, and human subsistence behavior in southern Africa's Cape Floral Region. *Palaeogeography, Palaeoclimatology, Palaeoecology* **306**(3–4), 219-227. (doi:10.1016/j.palaeo.2011.04.025).

7. Cain J.W., Krausman P.R., Germaine H.L. 2004 *Antidorcas marsupialis*. *Mammalian Species*, 1-7. (doi:10.1644/753).

8. Klein R.G., Cruz-Uribe K., Beaumont P.B. 1991 Environmental, ecological, and paleoanthropological implications of the late Pleistocene mammalian fauna from Equus Cave, northern Cape Province, South Africa. *Quaternary Research* **36**(1), 94-119. (doi:10.1016/0033-5894(91)90019-2).

9. Ubilla M., Perea D., Goso Aguilar C., Lorenzo N. 2004 Late Pleistocene vertebrates from northern Uruguay: tools for biostratigraphic, climatic and environmental reconstruction. *Quaternary International* **114**(1), 129-142. (doi:10.1016/s1040-6182(03)00048-x).

10. Pereira J.C., Lopes R.P., Kerber L. 2012 New remains of Late Pleistocene mammals from Chuí creek, Southern Brazil. *Revista Brasileira de Paleontologia* **15**(2), 228-239.

11. Ferrusquía-Villafranca I., Arroyo-Cabrales J., Martínez-Hernández E., Gama-Castro J., Ruiz-González J., Polaco O.J., Johnson E. 2010 Pleistocene mammals of Mexico: A critical review of regional chronofaunas, climate change response and biogeographic provinciality. *Quaternary International* **217**(1–2), 53-104. (doi:10.1016/j.quaint.2009.11.036).

12. Soibelzon L.H., Tonni E.P., Bond M. 2005 The fossil record of South American short-faced bears (Ursidae, Tremarctinae). *Journal of South American Earth Sciences* **20**(1-2), 105-113. (doi:DOI: 10.1016/j.jsames.2005.07.005).

13. Prevosti F.J., Vizcaino S.F. 2006 Paleoecology of the large carnivore guild from the late Pleistocene of Argentina. *Acta Palaeontologica Polonica* **51**(3), 407-422.

14. Soibelzon L.H., Rincón A.D. 2007 The fossil record of the short-faced bears (Ursidae, Tremarctinae) from Venezuela. Systematic, biogeographic, and paleoecological implications. *Neues Jahrbuch für Geologie und Paläontologie-Abhandlungen* **244**(3), 287-298. (doi:10.1127/0077-7749/2007/0244-0287).

15. García López D.A., Ortiz P.E., Jaén M.C.M., Moyano M.S. 2008 First record of *Arctotherium* (Ursidae, Tremarctinae) in Northwestern Argentina and its paleobiogeographic significance. *Journal of Vertebrate Paleontology* **28**(4), 1232-1237. (doi:10.1671/0272-4634-28.4.1232).

16. Orlova L., Kuzmin Y.V., Dementiev V.N. 2004 *A review of the evidence for extinction chronologies for five species of Upper Pleistocene megafauna in Siberia*. Tucson, AZ, University of Arizona; 14 p.

17. Kalke H.D. 1986 Southern limits of the Late Pleistocene Euro-Siberian faunal complex in East Asia. In *Beringia in the cenozoic era* (eds. Kontrimavicius V.L., Balkema A.A.), pp. 346-358. Rotterdam: Balkema, Amerind Publishing Company.

18. Markova A.K., Puzachenko A.Y., van Kolfschoten T. 2010 The North Eurasian mammal assemblages during the end of MIS 3 (Brianskian–Late Karginian–Denekamp Interstadial). *Quaternary International* **212**(2), 149-158. (doi:10.1016/j.quaint.2009.02.010).

19. MacPhee R.D.E., Tikhonov A.N., Mol D., de Marliave C., van der Plicht H., Greenwood A.D., Flemming C., Agenbroad L. 2002 Radiocarbon chronologies and extinction dynamics of the Late Quaternary mammalian megafauna of the Taimyr Peninsula, Russian Federation. *Journal of Archaeological Science* **29**(9), 1017-1042. (doi:10.1006/jasc.2001.0802).

20. Burns J.A. 2010 Mammalian faunal dynamics in Late Pleistocene Alberta, Canada. *Quaternary International* **217**(1–2), 37-42. (doi:10.1016/j.quaint.2009.08.003).

21. Barnosky A.D., Koch P.L., Feranec R.S., Wing S.L., Shabel A.B. 2004 Assessing the causes of Late Pleistocene extinctions on the continents. *Science* **306**(5693), 70-75.

22. Roberts R., Flannery T., Ayliffe L., Yoshida H., Olley J., Prideaux G., Laslett G., Baynes A., Smith M., Jones R.I., et al. 2001 New ages for the last Australian megafauna: Continent-wide extinction about 46,000 years ago. *Science* **292**, 1888-1892.

23. Prideaux G.J., Gully G.A., Couzens A.M.C., Ayliffe L.K., Jankowski N.R., Jacobs Z., Roberts R.G., Hellstrom J.C., Gagan M.K., Hatcher L.M. 2010 Timing and dynamics of Late Pleistocene mammal extinctions in southwestern Australia. *P Natl Acad Sci USA* **107**(51), 22157-22162. (doi:10.1073/pnas.1011073107).

24. Johnson C. 2006 *Australia's Mammal Extinctions A 50000 year history*. Cambridge, Cambridge University Press.

25. van Vuure C. 2005 *Retracing the aurochs: history, morphology and ecology of an extinct wild ox*. Sofia-Moscow, Pensoft Publishers.

26. Dobson M. 2002 Mammal distributions in the western Mediterranean: the role of human intervention. *Mammal Review* **28**(2), 77-88.

27. Turvey S.T., Fritz S.A. 2011 The ghosts of mammals past: biological and geographical patterns of global mammalian extinction across the Holocene. *Philosophical Transactions of the Royal Society B: Biological Sciences* **366**(1577), 2564-2576. (doi:10.1098/rstb.2011.0020).

28. Hooijer D.A. 1958 Fossil Bovidae from the Malay archipelago and the Punjab. *Zoologische Verhandelingen van de Museum Leiden* **38**, 1-112.

29. van den Bergh G.D., de Vos J., Sondaar P.Y. 2001 The Late Quaternary palaeogeography of mammal evolution in the Indonesian Archipelago. *Palaeogeography, Palaeoclimatology, Palaeoecology* **171**(3-4), 385-408. (doi:10.1016/s0031-0182(01)00255-3).

30. Cartelle C., Hartwig W.C. 1996 A new extinct primate among the Pleistocene megafauna of Bahia, Brazil. *Proceedings of the National Academy of Sciences of the United States of America* **93**(13), 6405-6409.

31. Zazula G.D., Turner D.G., Ward B.C., Bond J. 2011 Last interglacial western camel (*Camelops hesternus*) from eastern Beringia. *Quaternary Sci Rev* **30**(19–20), 2355-2360. (doi:10.1016/j.quascirev.2011.06.010).

32. Spassov N., Stoytchev T. 2004 The dromedary domestication problem: 3000 BC rock art evidence for the existence of wild one-humped camel in Central Arabia. *Historia naturalis bulgarica* **16**, 151-158.

33. Ripinsky M. 1985 The Camel in Dynastic Egypt. *The Journal of Egyptian Archaeology* **71**, 134-141.

34. Mikesell M.W. 1955 Notes on the dispersal of the dromedary. *Southwestern Journal of Anthropology* **11**(3), 231-245.

35. Dundas R.G. 1999 Quaternary records of the dire wolf, *Canis dirus*, in North and South America. *Boreas* **28**(3), 375-385. (doi:10.1111/j.1502-3885.1999.tb00227.x).

36. Prevosti F., Tonni E., Bidegain J. 2009 Stratigraphic range of the large canids (Carnivora, Canidae) in South America, and its relevance to quaternary biostratigraphy. *Quaternary International* **210**(1), 76-81.

37. Faith J.T. 2014 Late Pleistocene and Holocene mammal extinctions on continental Africa. *Earth-Science Reviews* **128**, 105-121.

38. White R.S., Morgan G. 2011 Capromeryx (Artiodactyla: Antilocapridae) from the Rancholabrean Tramperos Creek fauna, Union County, New Mexico, with a review of the occurence and paleobiology of Capromeryx in the Rancholabrean of New Mexico. In *Fossil Record 3 New Mexico Muesum of Natural History and Science, Bulletin 53* (ed. al. S.e.).

39. Paleobiology database. 2012 http://paleodb.org. (

40. Russell D.A., Rich F.J., Schneider V., Lynch-Stieglitz J. 2009 A warm thermal enclave in the Late Pleistocene of the South-eastern United States. *Biological Reviews* **84**(2), 173-202. (doi:10.1111/j.1469-185X.2008.00069.x).

41. Kerber L., Kinoshita A., José F.A., Graciano Figueiredo A.M., Oliveira É.V., Baffa O. 2011 Electron Spin Resonance dating of the southern Brazilian Pleistocene mammals from Touro Passo Formation, and remarks on the geochronology, fauna and palaeoenvironments. *Quaternary International* **245**(2), 201-208. (doi:10.1016/j.quaint.2010.10.010).

42. Gasparini G. 2011 Records and stratigraphical ranges of South American Tayassuidae (Mammalia, Artiodactyla). *Journal of Mammalian Evolution*, 1-12. (doi:10.1007/s10914-011-9172-z).

43. Auler A.S., Piló L.B., Smart P.L., Wang X., Hoffmann D., Richards D.A., Edwards R.L., Neves W.A., Cheng H. 2006 U-series dating and taphonomy of Quaternary vertebrates from Brazilian caves. *Palaeogeography, Palaeoclimatology, Palaeoecology* **240**(3-4), 508-522. (doi:10.1016/j.palaeo.2006.03.002).

44. Ghilardi A.M., Fernandes M.A., Bichuette M.E. 2011 Megafauna from the Late Pleistocene-Holocene deposits of the Upper Ribeira karst area, southeast Brazil. *Quaternary International* **245**(2), 369-378. (doi:10.1016/j.quaint.2011.04.018).

45. Lopes R.P., Pereira J.C. 2010 Fossils of Scelidotheriinae Ameghino, 1904 (Xenarthra, Pilosa) in the Pleistocene deposits of Rio Grande do Sul, Brazil. *Gaea, Journal of Geoscience* **6**, 44-52.

46. Cartelle C., De Iuliis G., Lopes Ferreira R. 2009 Systematic revision of tropical Brazilian *Scelidotheriine* sloths (Xenarthra, Mylodontoidea). *Journal of Vertebrate Paleontology* **29**(2), 555-566. (doi:10.1671/039.029.0231).

47. Kurtén B. 1968 *Pleistocene mammals of Europe*. London, AldineTransaction.

48. Louys J., Curnoe D., Tong H. 2007 Characteristics of Pleistocene megafauna extinctions in Southeast Asia. *Palaeogeography, Palaeoclimatology, Palaeoecology* **243**(1–2), 152-173. (doi:10.1016/j.palaeo.2006.07.011).

49. Gonzalez S.V. 2011 Aplicación de los modelos de distribución de especies hacia el pasado. Análisis de la distribución y extinción de las poblaciones europeas de hiena manchada (*Crocuta crocuta* (Erxleben, 1777)) durante el Pleistoceno. Madrid, Universidad Autonoma De Madrid.

50. Prasad K.N. 1996 Pleistocene cave fauna from peninsular India. *Journal of Caves and Karst Studies* **58**(1), 30-34.

51. Mashkour M., Monchot H., Trinkaus E., Reyss J.L., Biglari F., Bailon S., Heydari S., Abdi K. 2009 Carnivores and their prey in the Wezmeh Cave (Kermanshah, Iran): a Late Pleistocene refuge in the Zagros. *International Journal of Osteoarchaeology* **19**(6), 678-694. (doi:10.1002/oa.997).

52. Stuart A.J., Lister A.M. 2013 New radiocarbon evidence on the extirpation of the spotted hyaena (*Crocuta crocuta* (Erxl.) in northern Eurasia. *Quaternary Sci Rev*.

53. Sommer R., Benecke N. 2005 Late-Pleistocene and early Holocene history of the canid fauna of Europe (Canidae). *Mammalian Biology - Zeitschrift für Säugetierkunde* **70**(4), 227-241. (doi:10.1016/j.mambio.2004.12.001).

54. Arroyo-Cabrales J., Johnson E., Haas H., De Los R.-P., Magdalena, Ralph R.W., Hartwell W.T. 1995 First Radiocarbon Dates for San Josecito Cave, Nuevo León, México. *Quaternary Research* **43**(2), 255-258. (doi:10.1006/qres.1995.1026).

55. Cisneros J.C. 2005 New Pleistocene vertebrate fauna from El Salvador. *Revista Brasileira de Paleontologia* **8**(3), 239-255.

56. Prado J.L., Alberdi M.T., Azanza B., Sánchez B., Frassinetti D. 2005 The Pleistocene Gomphotheriidae (Proboscidea) from South America. *Quaternary International* **126–128**(0), 21-30. (doi:10.1016/j.quaint.2004.04.012).

57. Arroyo-Cabrales J., Polaco O.J., Laurito C., Johnson E., Teresa Alberdi M., Valerio Zamora A.L. 2007 The proboscideans (Mammalia) from Mesoamerica. *Quaternary International* **169–170**(0), 17-23. (doi:10.1016/j.quaint.2006.12.017).

58. Barnosky A.D., Lindsey E.L. 2010 Timing of Quaternary megafaunal extinction in South America in relation to human arrival and climate change. *Quaternary International* **217**(1–2), 10-29. (doi:10.1016/j.quaint.2009.11.017).

59. Faith J.T., Choiniere J.N., Tryon C.A., Peppe D.J., Fox D.L. 2011 Taxonomic status and paleoecology of *Rusingoryx atopocranion* (Mammalia, Artiodactyla), an extinct Pleistocene bovid from Rusinga Island, Kenya. *Quaternary Research* **75**(3), 697-707. (doi:DOI: 10.1016/j.yqres.2010.11.006).

60. Faith J.T., Potts R., Plummer T.W., Bishop L.C., Marean C.W., Tryon C.A. 2012 New perspectives on middle Pleistocene change in the large mammal faunas of East Africa: *Damaliscus hypsodon* sp. nov. (Mammalia, Artiodactyla) from Lainyamok, Kenya. *Palaeogeography, Palaeoclimatology, Palaeoecology* **361–362**(0), 84-93. (doi:10.1016/j.palaeo.2012.08.005).

61. Rincón A.D., White R.S., McDonald H.G. 2008 Late Pleistocene Cingulates (Mammalia: Xenarthra) from Mene De Inciarte Tar Pits, Sierra De Perijá, Western Venezuela. *Journal of Vertebrate Paleontology* **28**(1), 197-207. (doi:10.1671/0272-4634(2008)28[197:lpcmxf]2.0.co;2).

62. Stewart J.R., Stringer C.B. 2012 Human evolution out of Africa: The role of refugia and climate change. *Science* **335**(6074), 1317-1321. (doi:10.1126/science.1215627).

63. Chauhan P.R. 2009 The lower Paleolithic of the Indian subcontinent. *Evolutionary Anthropology: Issues, News, and Reviews* **18**(2), 62-78.

64. Wood B., Collard M. 1999 The Human Genus. *Science* **284**(5411), 65-71. (doi:10.1126/science.284.5411.65).

65. Shockey B.J., Salas-Gismondi R., Baby P., Guyot J.-L., Baltazar M.C., Huamán L., Clack A.A., Stucchi M., Pujos F., Emerson J.M., et al. 2009 New Pleistocene cave faunas of the Andes of Central Peru: Radiocarbon ages and the survival of low latitude, Pleistocene DNA. *Palaeontologia Electronica* **12**(3), 15A-15p.

66. Pujos F., De Iuliis G., Argot C., Werdelin L. 2007 A peculiar climbing Megalonychidae from the Pleistocene of Peru and its implication for sloth history. *Zoological Journal of the Linnean Society* **149**(2), 179-235. (doi:10.1111/j.1096-3642.2007.00240.x).

67. Hubbe A., Hubbe M., Neves W. 2007 Early Holocene survival of megafauna in South America. *Journal of Biogeography* **34**(9), 1642-1646. (doi:10.1111/j.1365-2699.2007.01744.x).

68. Steele J., Politis G. 2009 AMS 14C dating of early human occupation of southern South America. *Journal of Archaeological Science* **36**(2), 419-429. (doi:10.1016/j.jas.2008.09.024).

69. Titov V.V., Tesakov A.S. 2010 Quaternary stratigraphy and paleontology of the southern Russia: connections between Europe, Africa and Asia. (p. 78. Rostov-on-Don, International Union for Quaternary Research, Section on European Quaternary Stratigraphy, Southern Scientific Centre, Russian Academy of Sciences Geological Institute.

70. Hagstrum J., Firestone R., West A. 2009 Beringian megafaunal extinctions at ~ 37 ka BP: Do micrometeorites embedded in fossil tusks and skulls indicate an extraterrestial precursor to the Younger Dryas event? In *AGU Fall Meeting Abstracts* (p. 1385.

71. Cerdeño E. 1998 Diversity and evolutionary trends of the Family Rhinocerotidae (Perissodactyla). *Palaeogeography, Palaeoclimatology, Palaeoecology* **141**(1–2), 13-34. (doi:10.1016/s0031-0182(98)00003-0).

72. Pushkina D. 2007 The Pleistocene easternmost distribution in Eurasia of the species associated with the Eemian *Palaeoloxodon antiquus* assemblage. *Mammal Review* **37**(3), 224-245. (doi:10.1111/j.1365-2907.2007.00109.x).

73. Albayrak E., Lister A.M. 2011 Dental remains of fossil elephants from Turkey. *Quaternary International* **276–277**(0), 198-211. (doi:10.1016/j.quaint.2011.05.042).

74. Mol D., de Vos J., van der Plicht J. 2007 The presence and extinction of *Elephas antiquus* Falconer and Cautley, 1847, in Europe. *Quaternary International* **169–170**(0), 149-153. (doi:10.1016/j.quaint.2006.06.002).

75. Göhlich U. 2000 On a pelvis of the straight-tusked elephant *Elephas antiquus* (Proboscidea, Mammalia) from Binsfeld near Speyer (Rhineland-Palatinate, Germany). *Paläontologische Zeitschrift* **74**(1), 205-214. (doi:10.1007/bf02987962).

76. Tsoukala E., Mol D., Pappa S., Vlachos E., van Logchem W., Vaxevanopoulos M., Reumer J. 2011 *Elephas antiquus* in Greece: New finds and a reappraisal of older material (Mammalia, Proboscidea, Elephantidae). *Quaternary International* **245**(2), 339-349. (doi:10.1016/j.quaint.2010.10.008).

77. Davies P., Lister A.M. 2001 Palaeoloxodon cypriotes, the dwarf elephant of Cyprus: size and scaling comparisons with P. falconeri (Sicily-Malta) and mainland P. antiquus. In *The World of Elephants - International Congress* (pp. 479-480. Rome.

78. Werdelin L., Sanders W.J. 2010 *Cenozoic Mammals of Africa*. Ewing, University of California Press.

79. Maglio V., Cooke H. 1978 Evolution of African Mammals. (Boston, Harvard University Press.

80. Todd N. 2006 Trends in Proboscidean Diversity in the African Cenozoic. *Journal of Mammalian Evolution* **13**(1), 1-10. (doi:10.1007/s10914-005-9000-4).

81. Mol D., van den Bergh G.D. 1999 Fossil proboscideans from the Netherlands, the North Sea and the Oosterschelde estuary. *Deinsea (Mammoths & the Mammoth Fauna)* **6**, 119-145.

82. Palombo M.R., Anzidei A.P., Arnoldus-Huyzendveld A. 2003 La Polledrara di Cecanibbio: one of the richest *Elephas* (Palaeoloxodon) *antiquus* sites of the late Middle Pleistocene in Italy. *Deinsea* **9**, 317-330.

83. Park Y.A., Yi H., II. 1995 Late Quaternary climatic changes and sea-level history along the Korean coasts. *Journal of Coastal Research* (Special Issue No. 17.), 163-168. (doi:10.2307/25735639).

84. Kondo Y., Mazima N. 1990 Palaeoloxodon naumanni and its environment at the Palaeolithic site of Lake Nojiri, Nagano Prefecture, Central Japan. In *The World of Elephants* (ed. G. Cavarretta P.G., M. Mussi & M.R. Palombo), pp. 284-288. Rome, CNR.

85. Sondaar P.Y., Van der Geer A.A.E. 2005 Evolution and extinction of Plio-Pleistocene island ungulates. In *Les ongulés holarctiques du Pliocène et du Pléistocene* (ed. Cregut-Bonnoure E.), pp. 241-256. Paris, Maison de la Géologie.

86. Li J., Hou Y., Li Y., Zhang J. 2012 The latest straight-tusked elephants (*Palaeoloxodon*)? “Wild elephants” lived 3000 years ago in North China. *Quaternary International* **281**(0), 84-88. (doi:10.1016/j.quaint.2011.10.039).

87. Morlan R.E. 1967 Chronometric dating in Japan. *Arctic Anthropology* **4**(2), 180-211. (doi:10.2307/40315649).

88. Bennett D., Hoffmann R.S. 1999 *Equus caballus*. *Mammalian Species* (628), 1-14.

89. Orlando L., Metcalf J.L., Alberdi M.T., Telles-Antunes M., Bonjean D., Otte M., Martin F., Eisenmann V., Mashkour M., Morello F., et al. 2009 Revising the recent evolutionary history of equids using ancient DNA. *Proceedings of the National Academy of Sciences of the United States of America* **106**(51), 21754-21759. (doi:10.1073/pnas.0903672106).

90. Weinstock J., Willerslev E., Sher A., Tong W., Ho S.Y.W., Rubenstein D., Storer J., Burns J., Martin L., Bravi C., et al. 2005 Evolution, Systematics, and Phylogeography of Pleistocene Horses in the New World: A Molecular Perspective. *PLoS Biology* **3**(8), e241.

91. Holanda E.C., Ferigolo J., Ribeiro A.M. 2011 New Tapirus species (Mammalia: Perissodactyla: Tapiridae) from the upper Pleistocene of Amazonia, Brazil. *Journal of Mammalogy* **92**(1), 111-120. (doi:10.1644/10-mamm-a-144.1).

92. Martinelli A.G., Ferraz P.F., Cunha G.C., Cunha I.C., de Souza Carvalho I., Borges Ribeiro L.C., Neto F.M., Cavellani C.L., de Paula Antunes Teixeira V., da Fonseca Ferraz M.L. 2012 First record of *Eremotherium laurillardi* (Lund, 1842) (Mammalia, Xenarthra, Megatheriidae) in the Quaternary of Uberaba, Triângulo Mineiro (Minas Gerais State), Brazil. *Journal of South American Earth Sciences* **37**(0), 202-207. (doi:10.1016/j.jsames.2012.03.006).

93. Cartelle C., Iuliis G.D. 1995 *Eremotherium laurillardi*: The panamerican Late Pleistocene megatheriid sloth. *Journal of Vertebrate Paleontology* **15**(4), 830-841.

94. Mazzanti D.L., Quintana C.A. 1997 Asociación cultural de fauna extinguida en el sitio arqueológico Cueva Tixi, provincia de Buenos Aires, Argentina. *Revista Española de Antropología Americana* **27**, 11.

95. McAfee R.K. 2009 Reassessment of the cranial characters of *Glossotherium* and *Paramylodon* (Mammalia: Xenarthra: Mylodontidae). *Zoological Journal of the Linnean Society* **155**(4), 885-903. (doi:10.1111/j.1096-3642.2008.00468.x).

96. Oliveira, dison V., Porpino K.O., Barreto A.F. 2010 On the presence of Glyptotherium in the Late Pleistocene of Northeastern Brazil, and the status of "*Glyptodon*" and "*Chlamydotherium*". Paleobiogeographic implications. *Neues Jahrbuch für Geologie und Paläontologie - Abhandlungen* **258**(3), 353-363. (doi:10.1127/0077-7749/2010/0116).

97. Baffa O., Brunetti A., Karmann I., Neto C.M.D. 2000 ESR dating of a toxodon tooth from a Brazilian karstic cave. *Applied Radiation and Isotopes* **52**(5), 1345-1349. (doi:10.1016/s0969-8043(00)00093-2).

98. Dantas M.A.T., França L.d.M., Cozzuol M.A., Rincón A.D. 2011 About the occurrence of *Glyptodon* sp. in the Brazilian intertropical region. *Quaternary International* (0). (doi:10.1016/j.quaint.2011.06.024).

99. Ribeiro R.d.C., Kinoshita A., Figueiredo A.M.G., Carvalho I.d.S., Baffa O. 2012 Electron Spin Resonance dating of the Late Quaternary megafauna fossils from Baixa Grande, Bahia, Brazil. *Quaternary International* (0). (doi:10.1016/j.quaint.2012.07.017).

100. Gillette D.D., Ray C.E. 1981 Glyptodonts of North America. *Smithsonian Contributions to Paleobiology* **40**.

101. Scherer C.S. 2012 The Camelidae (Mammalia, Artiodactyla) from the Quaternary of South America: Cladistic and biogeographic hypotheses. *Journal of Mammalian Evolution*, 1-12. (doi:10.1007/s10914-012-9203-4).

102. Rivals F., Blasco R. 2008 Presence of *Hemitragus* aff. *cedrensis* (Mammalia, Bovidae) in the Iberian Peninsula: Biochronological and biogeographical implications of its discovery at Bolomor Cave (Valencia, Spain). *Comptes Rendus Palevol* **7**(6), 391-399. (doi:10.1016/j.crpv.2008.05.003).

103. Szmidt C.C., Moncel M.-H., Daujeard C. 2010 New data on the Late Mousterian in Mediterranean France: First radiocarbon (AMS) dates at Saint-Marcel Cave (Ardèche). *Comptes Rendus Palevol* **9**(4), 185-199. (doi:10.1016/j.crpv.2010.05.002).

104. Rivals F. 2004 *Les petits bovidés (Caprini et Rupicaprini) pléistocènes dans le bassin méditerranéen et le Caucase. Etude paléontologique, biostratigraphique, archéozoologique et paléoécologique.* Oxford, Archaeopress.

105. Boisserie J.-R., White T.D. 2004 A new species of Pliocene Hippopotamidae from the Middle Awash, Ethiopia. *Journal of Vertebrate Paleontology* **24**(2), 464-473.

106. Alberdi M.T., Prado J.L. 1998 Comments on: Pleistocene horses from Tarija, Bolivia, and validity of the genus *Onohippidium* (Mammalia: Equidae), by B. J. MacFadden. *Journal of Vertebrate Paleontology* **18**(3), 669-672. (doi:10.1080/02724634.1998.10011094).

107. Ham R.W.J.M., Kuijper W.J., Kortselius M.J.H., Burgh J., Stone G.N., Brewer J.G. 2008 Plant remains from the Kreftenheye Formation (Eemian) at Raalte, The Netherlands. *Veget Hist Archaeobot* **17**(1), 127-144. (doi:10.1007/s00334-007-0115-9).

108. Currant A., Jacobi R. 2001 A formal mammalian biostratigraphy for the Late Pleistocene of Britain. *Quaternary Sci Rev* **20**(16–17), 1707-1716. (doi:10.1016/s0277-3791(01)00035-x).

109. Scillato-Yané G.J., Carlini A.A., Tonni E.P., Noriega J.I. 2005 Paleobiogeography of the late Pleistocene pampatheres of South America. *Journal of South American Earth Sciences* **20**(1-2), 131-138. (doi:10.1016/j.jsames.2005.06.012).

110. Swisher C.C., Rink W.J., Antón S.C., Schwarcz H.P., Curtis G.H., Suprijo A., Widiasmoro. 1996 Latest *Homo erectus* of Java: potential contemporaneity with *Homo sapiens* in southeast Asia. *Science* **274**(5294), 1870-1874.

111. Mol D., Post K., Reumer J.W.F., van der Plicht J., de Vos J., van Geel B., van Reenen G., Pals J.P., Glimmerveen J. 2006 The Eurogeul—first report of the palaeontological, palynological and archaeological investigations of this part of the North Sea. *Quaternary International* **142–143**(0), 178-185. (doi:10.1016/j.quaint.2005.03.015).

112. van Weers D.J. 2005 A taxonomic revision the Pleistocene Hystrix (Hystricidae, Rodentia) from Eurasia with notes on the evolution of the family. *Contributions to Zoology* **74**(3/4), 301-312.

113. Tong H. 2008 Quaternary *Hystrix* (Rodentia, Mammalia) from North China: Taxonomy, stratigraphy and zoogeography, with discussions on the distribution of Hystrix in Palearctic Eurasia. *Quaternary International* **179**(1), 126-134. (doi:10.1016/j.quaint.2007.09.003).

114. Wu X., Liu W., Gao X., Yin G. 2006 Huanglong cave, a new late pleistocene hominid site in Hubei Province, China. *Chinese Science Bulletin* **51**(20), 2493-2499. (doi:10.1007/s11434-006-2125-x).

115. Fooden J. 2007 Systematic review of the Barbary Macaque, *Macaca sylvanus* (Linnaeus, 1758). *Fieldiana, Zoology* (113), 1-32.

116. Castaños P., Murelaga X., Arrizabalaga A., Iriarte M.J. 2011 First evidence of *Macaca sylvanus* (Primates, Cercopithecidae) from the Late Pleistocene of Lezetxiki II cave (Basque Country, Spain). *Journal of Human Evolution* **60**(6), 816.

117. Ferrero B., Brandoni D., Noriega J.I., Carlini A.A. 2007 Mamíferos de la Formación El Palmar (Pleistoceno tardío) de la provincia de Entre Ríos, Argentina. *Revista del Museo* **9**(2), 109-117.

118. Scherer C.S., Pitana V.G., Ribeiro A.M. 2009 Proterotheriidae and Macraucheniidae (Liptoterna, Mammalia) from the Pleistocene of Rio Grande do Sul State, Brazil. *Revista Brasileira de Paleontologia* **12**(3), 231-246.

119. Raia P., Carotenuto F., Meiri S. 2010 One size does not fit all: no evidence for an optimal body size on islands. *Global Ecology and Biogeography* **19**(4), 475-484. (doi:10.1111/j.1466-8238.2010.00531.x).

120. Álvarez-Lao D.J., García N. 2011 Geographical distribution of Pleistocene cold-adapted large mammal faunas in the Iberian Peninsula. *Quaternary International* **233**(2), 159-170. (doi:10.1016/j.quaint.2010.04.017).

121. Sommer R.S., Nadachowski A. 2006 Glacial refugia of mammals in Europe: evidence from fossil records. *Mammal Review* **36**(4), 251-265. (doi:10.1111/j.1365-2907.2006.00093.x).

122. Kuzmin Y.V., Orlova L.A. 2004 Radiocarbon chronology and environment of woolly mammoth (*Mammuthus primigenius* Blum.) in northern Asia: results and perspectives. *Earth-Science Reviews* **68**(1–2), 133-169. (doi:10.1016/j.earscirev.2004.04.002).

123. Debruyne R., Chu G., King C.E., Bos K., Kuch M., Schwarz C., Szpak P., Gröcke D.R., Matheus P., Zazula G., et al. 2008 Out of America: Ancient DNA evidence for a New World origin of Late Quaternary woolly mammoths. *Current Biology* **18**(17), 1320-1326. (doi:10.1016/j.cub.2008.07.061).

124. Boeskorov G.G. 2006 Arctic Siberia: refuge of the Mammoth fauna in the Holocene. *Quaternary International* **142–143**(0), 119-123. (doi:10.1016/j.quaint.2005.03.009).

125. Stuart A.J. 2005 The extinction of woolly mammoth (*Mammuthus primigenius*) and straight-tusked elephant (*Palaeoloxodon antiquus*) in Europe. *Quaternary International* **126–128**(0), 171-177. (doi:10.1016/j.quaint.2004.04.021).

126. Majid Z. 2005 *The Perak man and other prehistoric skeletons of Malaysia*. Pulau Pinang, Penerbit Universiti Sains Malaysia.

127. Lane D. 2010 Late Quaternary turnover of mammals in Borneo: the zooarchaeological record. In *Tropical islands biodiversity crisis: the Indo-West Pacific A conference organized by the Universiti Brunei Darussalam, Gadong, Brunei, 11-13 June 2007* (pp. 373-391, Springer.

128. Norman L. 2008 Autecology of the sunda pangolin (Manis javanica) in Singapore. Singapore, National University of Singapore.

129. Turney C.S.M., Flannery T.F., Roberts R.G., Reid C., Fifield L.K., Higham T.F.G., Jacobs Z., Kemp N., Colhoun E.A., Kalin R.M., et al. 2008 Late-surviving megafauna in Tasmania, Australia, implicate human involvement in their extinction. *Proceedings of the National Academy of Sciences of the United States of America* **105**(34), 12150-12153. (doi:10.1073/pnas.0801360105).

130. Abbazzi L. 2004 Remarks on the validity of the generic name *Praemegaceros* portis 1920, and an overview on *Praemegaceros* species in Italy. *Rendiconti Lincei* **15**(2), 115-132. (doi:10.1007/bf02904712).

131. Hadjouis D. 1990 *Megaceroides algericus* (Lydekker, 1890), du gisement des Phacochères (Alger, Algérie). Etude critique de la position systématique de Megaceroides. *Quaternaire*, 247-258.

132. Merzoug S., Sari L. 2008 Re-examination of the Zone I material from Tamar Hat (Algeria): Zooarchaeological and technofunctional analyses. *African Archaeological Review* **25**(1), 57-73. (doi:10.1007/s10437-008-9028-y).

133. Vislobokova I. 2011 Historical development and geographical distribution of giant deer (*Cervidae*, Megacerini). *Paleontological Journal* **45**(6), 674-688. (doi:10.1134/s0031030111060153).

134. Olszewski D., Schurmans U., Schmidt B. 2011 The Epipaleolithic (Iberomaurusian) from Grotte des Contrebandiers, Morocco. *African Archaeological Review* **28**(2), 97-123. (doi:10.1007/s10437-010-9086-9).

135. Stuart A.J., Kosintsev P., Higham T., Lister A. 2004 Pleistocene to Holocene extinction dynamics in giant deer and woolly mammoth. *Nature* **431**(7009), 684-689.

136. Gao X., Huang W., Xu Z., Ma Z., Olsen J.W. 2004 120–150 ka human tooth and ivory engravings from Xinglongdong Cave, Three Gorges Region, South China. *Chinese Science Bulletin* **49**(2), 175-180. (doi:10.1360/03wd0214).

137. De Iuliis G., Pujos F., Tito G. 2009 Systematic and taxonomic revision of the Pleistocene ground sloth *Megatherium* (Pseudomegatherium) *tarijense* (Xenarthra: Megatheriidae). *Journal of Vertebrate Paleontology* **29**(4), 1244-1251. (doi:10.1671/039.029.0426).

138. Iuliis G.D., Pujos F., Tito G. 2009 Systematic and Taxonomic Revision of the Pleistocene Ground Sloth Megatherium (Pseudomegatherium) Tarijense (Xenarthra: Megatheriidae). *Journal of Vertebrate Paleontology* **29**(4), 1244-1251. (doi:10.1671/039.029.0426).

139. Prideaux G.J. 2004 *Systematics and evolution of the sthenurine kangaroo*. Ewing, University of California Press.

140. Cruz-Uribe K. 1983 The mammalian fauna from Redcliff Cave, Zimbabwe. *The South African Archaeological Bulletin* **38**(137), 7-16.

141. MacFadden B.J. 2005 Diet and habitat of toxodont megaherbivores (Mammalia, Notoungulata) from the late Quaternary of South and Central America. *Quaternary Research* **64**(2), 113-124. (doi:10.1016/j.yqres.2005.05.003).

142. Baskin J.A. 2005 Carnivora from the late Miocene Love Bone Bed of Florida. *Bulletin of the Florida Museum of Natural History* **45**(4), 413-434.

143. Mones A. 1991 Monografía de la familia Hydrochoeridae (Mammalia: Rodentia). *Courier Forschungsinstitut Senckenberg* **134**.

144. Oliveira E.V., Kerber L. 2009 Paleontologia e aspectos geológicos das sucessões do fi nal do Neógeno no sudoeste do Rio Grande do Sul, Brasil. *Journal of Geoscience* **5**(1), 21-34.

145. Ubilla M., Perea D., Bond M., Rinderknecht A. 2011 The first cranial remains of the Pleistocene proterotheriid Neolicaphrium recens Frenguelli, 1921 (Mammalia Litopterna): a comparative approach. *Journal of Vertebrate Paleontology* **31**, 193-201.

146. Zurita A.E., Ferrero B.S. 2009 Una nueva especie de Neuryurus Ameghino (Mammalia, Glyptodontidae) en el Pleistoceno tardío de la Mesopotamia de Argentina. *Geobios-Lyon* **42**(5), 663-673. (doi:10.1016/j.geobios.2009.03.003).

147. Vizcaıno S.F., Cassini G.H., Fernicola J.C., Bargo M.S. 2011 Evaluating habitats and feeding habits through ecomorphological features in glyptodonts (Mammalia, Xenarthra). *Ameghiniana* **48**, 305-319.

148. Steadman D.W., Martin P.S., MacPhee R.D.E., Jull A.J.T., McDonald H.G., Woods C.A., Iturralde-Vinent M., Hodgins G.W.L. 2005 Asynchronous extinction of late Quaternary sloths on continents and islands. *P Natl Acad Sci USA* **102**(33), 11763-11768. (doi:10.1073/pnas.0502777102).

149. Thompson R.S., Van Devender T.R., Martin P.S., Foppe T., Long A. 1980 Shasta ground sloth (*Nothrotheriops shastense* hoffstetter) at Shelter Cave, New Mexico: Environment, diet, and extinction. *Quaternary Research* **14**(3), 360-376. (doi:10.1016/0033-5894(80)90017-4).

150. Mead J.I., O'Rourke M.K., Foppe T.M. 1986 Dung and Diet of the Extinct Harrington's Mountain Goat (Oreamnos harringtoni). *Journal of Mammalogy* **67**(2), 284-293. (doi:10.2307/1380881).

151. Mead J.I., Agenbroad L.D., Phillips Iii A.M., Middleton L.T. 1987 Extinct mountain goat (*Oreamnos harringtoni*) in Southeastern Utah. *Quaternary Research* **27**(3), 323-331. (doi:10.1016/0033-5894(87)90087-1).

152. Markova A.K., Simakova A.N., Puzachenko A.Y. 2009 Ecosystems of Eastern Europe at the time of maximum cooling of the Valdai glaciation (24–18 kyr BP) inferred from data on plant communities and mammal assemblages. *Quaternary International* **201**(1–2), 53-59. (doi:10.1016/j.quaint.2008.05.020).

153. Stewart J.R. 2007 Neanderthal extinction as part of the faunal change in Europe during Oxygen Isotope Stage 3. *Acta Zoologica Cracoviensia - Series A: Vertebrata* **50**(1-2), 93-124. (doi:10.3409/000000007783995372).

154. Faure M., Guérin C., Parenti F. 1999 Découverte d'une mégafaune holocène à la Toca do Serrote do Artur (aire archéologique de São Raimundo Nonato, Piauî, Brésil): A gruta do Serrote do Artur (área arqueológica de São Raimundo Nonato, Piaúi, Brasil): dataçoes holocênicas para megafáuna de mamíferos. *Comptes Rendus de l'Académie des Sciences - Series IIA - Earth and Planetary Science* **329**(6), 443-448. (doi:10.1016/s1251-8050(00)80069-5).

155. Coltorti M., Abbazzi L., Ferretti M.P., Iacumin P., Rios F.P., Pellegrini M., Pieruccini P., Rustioni M., Tito G., Rook L. 2007 Last Glacial mammals in South America: a new scenario from the Tarija Basin (Bolivia). *Naturwissenschaften* **94**(4), 288-299. (doi:10.1007/s00114-006-0196-9).

156. Barnett R., Shapiro B., Barnes I.A.N., Ho S.Y.W., Burger J., Yamaguchi N., Higham T.F.G., Wheeler H.T., Rosendahl W., Sher A.V., et al. 2009 Phylogeography of lions (*Panthera leo* ssp.) reveals three distinct taxa and a late Pleistocene reduction in genetic diversity. *Molecular Ecology* **18**(8), 1668-1677. (doi:10.1111/j.1365-294X.2009.04134.x).

157. Sommer R.S., Benecke N. 2006 Late Pleistocene and Holocene development of the felid fauna (Felidae) of Europe: a review. *J Zool* **269**(1), 7-19. (doi:10.1111/j.1469-7998.2005.00040.x).

158. Stuart A.J., Lister A.M. 2011 Extinction chronology of the cave lion *Panthera spelaea*. *Quaternary Sci Rev* **30**(17–18), 2329-2340. (doi:10.1016/j.quascirev.2010.04.023).

159. McDonald H.G., Agenbroad L.D., Haden C.M., Jones C.A. 2004 Late Pleistocene mylodont sloth *Paramylodon harlani* (Mammalia: Xenarthra) from Arizona. *The Southwestern Naturalist* **49**(2), 229-238. (doi:10.1894/0038-4909(2004)049<0229:lpmsph>2.0.co;2).

160. Faith J.T. 2012 Palaeozoological insights into management options for a threatened mammal: southern Africa’s Cape mountain zebra (*Equus zebra zebra*). *Diversity and Distributions* **18**(5), 438-447. (doi:10.1111/j.1472-4642.2011.00841.x).

161. Klein R.G. 1994 The long-horned African buffalo (*Pelorovis antiquus*) is an extinct species. *Journal of Archaeological Science* **21**(6), 725-733. (doi:DOI: 10.1006/jasc.1994.1072).

162. Cupper M.L., Duncan J. 2006 Last glacial megafaunal death assemblage and early human occupation at Lake Menindee, southeastern Australia. *Quaternary Research* **66**(2), 332-341. (doi:10.1016/j.yqres.2006.06.004).

163. Price G.J., Webb G.E., Zhao J.-x., Feng Y.-x., Murray A.S., Cooke B.N., Hocknull S.A., Sobbe I.H. 2011 Dating megafaunal extinction on the Pleistocene Darling Downs, eastern Australia: the promise and pitfalls of dating as a test of extinction hypotheses. *Quaternary Sci Rev* **30**(7–8), 899-914. (doi:10.1016/j.quascirev.2011.01.011).

164. Flannery T.F. 1983 Quaternary kangaroos (Macropodidae: Marsupialia) from Nombe Rock Shelter, Papua New Guinea, with comments on the nature of megafaunal extinction in the New Guinea Highlands. *Proceedings of the Linnean Society of New South Wales* **107**, 75-97.

165. Harington C.R., Cinq-Mars J. 1995 Radiocarbon dates on saiga antelope (*Saiga tatarica*) fossils from Yukon and the Northwest Territories. *Arctic* **48**(1), 1-7.

166. Kurtén B. 1979 The stilt-legged deer *Sangamona* of the North-American Pleistocene. *Boreas* **8**(3), 313-321. (doi:10.1111/j.1502-3885.1979.tb00815.x).

167. Cruz L.E., Bargo M.S., Tonni E.P., Figini A.J. 2010 Radiocarbon date on megafauna from the late Pleistocene-early Holocene of Córdoba province, Argentina: stratigraphic and paleoclimatic significance. *Revista Mexicana de Ciencias Geológicas* **27**(3), 470-476.

168. Zurita A., Scarano A., Carlini A., Scillato-Yane G., Soibelzon E. 2011 *Neosclerocalyptus* spp.(Cingulata: Glyptodontidae: Hoplophorini): cranial morphology and palaeoenvironments along the changing Quaternary. *Journal of Natural History* **45**(15-16), 893-914.

169. van der Made J., Tong H.W. 2008 Phylogeny of the giant deer with palmate brow tines *Megaloceros* from west and *Sinomegaceros* from east Eurasia. *Quaternary International* **179**(1), 135-162. (doi:10.1016/j.quaint.2007.08.017).

170. Iwase A., Hashizume J., Izuho M., Takahashi K., Sato H. 2012 Timing of megafaunal extinction in the late Late Pleistocene on the Japanese Archipelago. *Quaternary International* **255**(0), 114-124. (doi:10.1016/j.quaint.2011.03.029).

171. Edmund A.G. 1965 *A late Pleistocene fauna from the Santa Elena Peninsula, Ecuador*. Toronto, Royal Ontario Museum.

172. Seymour K. 2010 The late Pleistocene fossil vertebrates from the Talara tar seeps, Peru, and Corralito, Ecuador, with particular reference to the Carnivora. In *X Congreso Argentino de Paleontología y Bioestratigrafía-VII Congreso Latinoamericano de Paleontología* (

173. Wei D., Qinqi X., Changzhu J., Jinyi L. 2010 The Quaternary herbivore faunas in North-east China, evolution under climate change. *Chinese Science Bulletin*, 129-132. (doi:http://dx.doi.org/10.1016/j.yqres.2010.11.006).

174. Khenzykhenova F.I. 2008 Paleoenvironments of Palaeolithic humans in the Baikal region. *Quaternary International* **179**(1), 53-57. (doi:10.1016/j.quaint.2007.09.004).

175. Dong W., Fu R., Feng X., Zhang S. 2009 Late Pleistocene mammalian fauna from the Mashandong, Chaoyang, Liaoning Province. *ACTA Anthropologica sinica* **1**, 011.

176. Junjie Y.X.M.Q.Y., Zhongming G. 1999 Liu Guanbang (Department of Earth Sciences, Nanjing University, Nanjing 210093); Pieces of pottery from Late Pleistocene Salawusu formation in Qingyang district, Gansu province [J]. *Geological Journal of China Universities* **1**.

177. Sato T., Khenzykhenova F. 2010 Mammoth fauna of Baikal Siberia: Results of contemporary archaeological studies. In *Quaternary stratigraphy and paleontology of the Southern Russia: connections between Europe, Africa and Asia* (p. 134.

178. Min L., Zhu G., Guan Y. 2009 An analysis of the basic characteristics of the Upper Pleistocene Salawusuan Stage in the Salawusu River Valley, Inner Mongolia. *Geology in China* **6**, 004.

179. Takahashi K., Wei G., Uno H., Yoneda M., Jin C., Sun C., Zhang S., Zhong B. 2007 AMS 14C chronology of the world's southernmost woolly mammoth (*Mammuthus primigenius* Blum.). *Quaternary Sci Rev* **26**(7–8), 954-957. (doi:10.1016/j.quascirev.2006.12.001).

180. Van Den Bergh G.D., Awe R.D., Morwood M.J., Sutikna T., Jatmiko, Wahyu Saptomo E. 2008 The youngest stegodon remains in Southeast Asia from the Late Pleistocene archaeological site Liang Bua, Flores, Indonesia. *Quaternary International* **182**(1), 16-48. (doi:http://dx.doi.org/10.1016/j.quaint.2007.02.001).

181. Encina R.L., Alberdi M.T. 2011 An updated taxonomic view on the family Gomphotheriidae (Proboscidea) in the final Pleistocene of south-central Chile. *Neues Jahrbuch für Geologie und Paläontologie - Abhandlungen* **262**(43-57).

182. Tong H., Wu X. 2010 *Stephanorhinus kirchbergensis* (Rhinocerotidae, Mammalia) from the Rhino Cave in Shennongjia, Hubei. *Chinese Science Bulletin* **55**(12), 1157-1168. (doi:10.1007/s11434-010-0050-5).

183. Billia E.M.E. 2011 Occurrences of *Stephanorhinus kirchbergensis* (Jager, 1839) (Mammalian, Rhinocerotidae) in Eurasia - An account. *Acta Palaeontologica Romaniae* **7**, 17-40.

184. Hardjasasmita H.S. 1987 Taxonomy and phylogeny of the Suidae (Mammalia) in Indonesia. *Scripta Geologica* **85**.

185. Otvos Jr E.G. 1980 Age of Tunica Hills (Louisiana-Mississippi) Quaternary fossiliferous creek deposits; Problems of radiocarbon dates and intermediate valley terraces in coastal plains. *Quaternary Research* **13**(1), 80-92. (doi:10.1016/0033-5894(80)90084-8).

186. Jefferson G.T. 1989 Late Cenozoic Tapirs (Mammalia: Perissodactyla) of western North America. *Natural History Museum of Los Angeles Cunty*.

187. Schubert B.W., Mead J.I., Graham R.W. 2003 *Ice Age Cave Faunas of North America*. Bloomington, Indiana University Press.

188. Price G., Webb G. 2006 Late Pleistocene sedimentology, taphonomy and megafauna extinction on the Darling Downs, southeastern Queensland. *Australian Journal of Earth Sciences* **53**(6), 947-970.

189. Knapp M., Rohland N., Weinstock J., Baryshnikov G., Sher A., Nagel D., Rabeder G., Pinhasi R.O.N., Schmidt H.A., Hofreiter M. 2009 First DNA sequences from Asian cave bear fossils reveal deep divergences and complex phylogeographic patterns. *Molecular Ecology* **18**(6), 1225-1238. (doi:10.1111/j.1365-294X.2009.04088.x).

190. Rabal-Garcés R., Cuenca-Bescós G., Ignacio Canudo J., De Torres T. 2012 Was the European cave bear an occasional scavenger? *Lethaia* **45**(1), 96-108.

191. Porpino K.D.O., Santos M., Bergqvist L.P. 2004 Registros de mamiferos fosseils no lajedo de soledade, Apodi, Rio Grande Do Norte, Brazil. *Revista Brasileira de Paleontologia* **7**(3), 349-358.

192. Wilson L.A.B., Sánchez-Villagra M.R., Madden R.H., Kay R.F. 2012 Testing a developmental model in the fossil record: molar proportions in South American ungulates. *Paleobiology* **38**(2), 308-321. (doi:10.1666/11001.1).
