# Supplementary material for: Global late Quaternary megafauna extinctions linked to humans, not climate change
Source: Proc Biol Sci. 2014 Jul 22;281(1787):20133254. doi: 10.1098/rspb.2013.3254 (PMC4071532; doi:10.1098/rspb.2013.3254)
Supplement: R analysis script [file rspb20133254supp5.rtf]

# Megafauna Analysis Script# Functions and Packages Script########################################### Load required Packages##########################################require("car")require("metafor")require("faraway")require("spdep")require("ncf")require("SpatialPack")########################################### Run required Functions##########################################trans.arcsine <- function(x){  asin(sign(x) * sqrt(abs(x)))}# see http://gettinggeneticsdone.blogspot.dk/2011/01/rstats-function-for-extracting-f-test-p.htmllmp <- function (modelobject) {  if (class(modelobject) != "lm") stop("Not an object of class 'lm' ")  f <- summary(modelobject)$fstatistic  p <- pf(f[1],f[2],f[3],lower.tail=F)  attributes(p) <- NULL  return(p)}########################################### Read in the data file Supplementary Data 2.csv##########################################D3 = read.csv("Supplementary Data 2.csv")D3$LargePropExtinctArcSine = trans.arcsine(D3$LargePropExtinctEDII)########################################### Run GLM analysis with arcsine square root transformation########################################### Climate only models# TempatureAnomClimate1 = lm(LargePropExtinctArcSine ~ MatAnomStand, data = D3)summary(Climate1)#PrecipAnomClimate2 = lm(LargePropExtinctArcSine ~ PrecipAnomStand,data = D3)summary(Climate2)#MatVelClimate3 = lm(LargePropExtinctArcSine ~ TempVelStand,data = D3)summary(Climate3)#PrecipVelClimate4 = lm(LargePropExtinctArcSine ~ PrecipVelStand,data = D3)summary(Climate4)#MatAnom +  PrecipAnomClimate5 = lm(LargePropExtinctArcSine ~ MatAnomStand + PrecipAnomStand,data = D3)summary(Climate5)#MatAnom *  PrecipAnomClimate6 = lm(LargePropExtinctArcSine ~ MatAnomStand * PrecipAnomStand,data = D3)summary(Climate6)#MatVel +  PrecipVelStandClimate7 = lm(LargePropExtinctArcSine ~ TempVelStand + PrecipAnomStand,data = D3)summary(Climate7)#MatVel +  PrecipVelStandClimate8 = lm(LargePropExtinctArcSine ~ TempVelStand * PrecipAnomStand,data = D3)summary(Climate8)#PrecipVelStand + MatAnomStandClimate9 = lm(LargePropExtinctArcSine ~ PrecipVelStand + MatAnomStand,data = D3)summary(Climate9)#PrecipVelStand * MatAnomStandClimate10 = lm(LargePropExtinctArcSine ~ PrecipVelStand * MatAnomStand,data = D3)summary(Climate10)#PrecipVelStand + TempVelStandClimate11 = lm(LargePropExtinctArcSine ~ PrecipVelStand + TempVelStand,data = D3)summary(Climate11)#PrecipVelStand * TempVelStandClimate12 = lm(LargePropExtinctArcSine ~ PrecipVelStand * TempVelStand,data = D3)summary(Climate12)nullModel = lm(LargePropExtinctArcSine ~ 1,data = D3)summary(nullModel)# Assess AIC's of each climate only modelClimateAICs = c(AIC(Climate1),AIC(Climate2),AIC(Climate3),AIC(Climate4),AIC(Climate5),AIC(Climate6),AIC(Climate7),AIC(Climate8),AIC(Climate9),AIC(Climate10),AIC(Climate11),AIC(Climate12))which(ClimateAICs == min(ClimateAICs)) #9summary(Climate9)# Model 9 has the lowest AIC that includes an interaction between PrecipVel and MatAnomClimate9 = lm(LargePropExtinctArcSine ~ MatAnomStand + PrecipVelStand, data = D3)Climate = summary(Climate9)[[4]]xmat = matrix(nrow = 7, ncol=4)xmat[1:3,1:4] = Climate[1:3,1:4]xmat[4,1] =  summary(Climate9)[[10]][1] #F statisticxmat[5,1] =  summary(Climate9)[[10]][3] # degrees of freedomxmat[6,1] =  summary(Climate9)[[9]] #Adjusted r squaredxmat[7,1] =  lmp(Climate9) #p valuexmat1 = data.frame(xmat)row.names(xmat1) = c("Intercept", "Temperature anomaly","Precipitation velocity","F statistic", "DoF","adjR2","p")names(xmat1) = c("Estimate", "Std. Error",   "t value",     "Pr(>|t|)")###########################################Human paleobiogeography only model########################################### Convert human paleobiogeography to numeric D3$HumanCat2 = NAD3$HumanCat2[which(D3$Human == "Modern Humans")] = 5D3$HumanCat2[which(D3$Human == "Pre-Sapiens peripheral")] = 4D3$HumanCat2[which(D3$Human == "Pre-Sapiens")] = 3D3$HumanCat2[which(D3$Human == "Archaic")] = 2D3$HumanCat2[which(D3$Human == "Homo Evolution")] = 1# Ordinal regressionmy.forward.diff = matrix(c(0.8, -0.2, -0.2, -0.2, -0.2, 0.6, 0.6, -0.4, -0.4, -0.4, 0.4, 0.4, 0.4, -0.6,-0.6, 0.2, 0.2, 0.2, 0.2, -0.8), ncol = 4)D3$HumanCat2 = as.factor(D3$HumanCat2)contrasts(D3$HumanCat2) = my.forward.diffHumanHis1 = lm(LargePropExtinctArcSine ~ HumanCat2, data = D3)summary(HumanHis1)#ordinal analysis of this model indicated there was no signifincat difference between levels 2 from 3 and 3 from 4 so use group 7# Set up human paleobiogeography with 3 regionsD3$HumanCat7 = as.character(D3$Human)D3$HumanCat7[which(D3$HumanCat7 == "Archaic" | D3$HumanCat7 == "Pre-Sapiens peripheral" | D3$HumanCat7 == "Pre-Sapiens")] = "ArcPrePre"D3$HumanCat7[which(D3$HumanCat7 == "Modern Humans")] = 3D3$HumanCat7[which(D3$HumanCat7 == "ArcPrePre")] = 2D3$HumanCat7[which(D3$HumanCat7 == "Homo Evolution")] = 1HumanHis7 = lm(LargePropExtinctArcSine ~ HumanCat7, data = D3)summary(HumanHis7)AIC(HumanHis7)Human = summary(HumanHis7)[[4]]xmat = matrix(nrow = 7, ncol=4)xmat[1:3,1:4] = Human[1:3,1:4]xmat[4,1] =  summary(HumanHis7)[[10]][1] #F statisticxmat[5,1] =  summary(HumanHis7)[[10]][3] # degrees of freedomxmat[6,1] =  summary(HumanHis7)[[9]] #Adjusted r squaredxmat[7,1] =  lmp(HumanHis7) #p valuexmat1 = data.frame(xmat)row.names(xmat1) = c("Intercept", "H. sapiens-only","Archaic-combined","F statistic", "DoF","adjR2","p")names(xmat1) = c("Estimate", "Std. Error",   "t value",     "Pr(>|t|)")###########################################Full model combining human paleobiogeography and climate variables###########################################Including all interactionsD3$HumanCat7 = as.factor(D3$HumanCat7)CombinedMod1 = lm(LargePropExtinctArcSine ~ HumanCat7  + MatAnomStand +  PrecipVelStand  + HumanCat7 * MatAnomStand + HumanCat7 * PrecipVelStand,data = D3)summary(CombinedMod1)#Not including the interaction between Human and Temperature anomalyCombinedMod2 = lm(LargePropExtinctArcSine ~ HumanCat7  + MatAnomStand +  PrecipVelStand + HumanCat7 * PrecipVelStand,data = D3)summary(CombinedMod2)#Not including the interaction between Human and Precipitation velocityCombinedMod3 = lm(LargePropExtinctArcSine ~HumanCat7  + MatAnomStand +  PrecipVelStand + HumanCat7 * MatAnomStand,data = D3)summary(CombinedMod3)#Not including temperature anomalyCombinedMod4 = lm(LargePropExtinctArcSine ~ HumanCat7  +  PrecipVelStand  +  HumanCat7 * PrecipVelStand,data = D3)summary(CombinedMod4)#Not including precipitation velocityCombinedMod5 = lm(LargePropExtinctArcSine ~ HumanCat7  +  MatAnomStand  +  HumanCat7 * MatAnomStand,data = D3)summary(CombinedMod5)#Only including Human and Temp AnomalyCombinedMod6 = lm(LargePropExtinctArcSine ~ HumanCat7  +  MatAnomStand,data = D3)summary(CombinedMod6)#Only including Human and Precip VelocityCombinedMod7 = lm(LargePropExtinctArcSine ~ HumanCat7  +  PrecipVelStand,data = D3)summary(CombinedMod7)CombinedAICAll = c(AIC(CombinedMod1),AIC(CombinedMod2),AIC(CombinedMod3),AIC(CombinedMod4),AIC(CombinedMod5),AIC(CombinedMod6),AIC(CombinedMod7))which(CombinedAICAll == min(CombinedAICAll))  #1#Model CombinedMod1 is the bestCombined = summary(CombinedMod1)[[4]]xmat = matrix(nrow = 13, ncol=4)xmat[1:9,1:4] = Combined[1:9,1:4]xmat[10,1] =  summary(CombinedMod1)[[10]][1] #F statisticxmat[11,1] =  summary(CombinedMod1)[[10]][3] # degrees of freedomxmat[12,1] =  summary(CombinedMod1)[[9]] #Adjusted r squaredxmat[13,1] =  lmp(CombinedMod1) #p valuexmat1 = data.frame(xmat)row.names(xmat1) = c("Intercept", "H. sapiens-only","Archaic-combined","Temperature anomaly","Precipitation velocity","HS x TA","AC x TA","HS x PV","AC x PV","F statistic", "DoF","adjR2","p")names(xmat1) = c("Estimate", "Std. Error",   "t value",     "Pr(>|t|)")summary(CombinedMod1)# Compare the combined model with the human only and climate onlymodel1 = anova(HumanHis7,CombinedMod5,test="Chisq")model1model2 = anova(Climate9,CombinedMod5,test="Chisq")model2########################################### Analysis using the family = quasi(link ="logit", variance = "mu(1-mu)")###########################################Null modelnullModel = glm(LargePropExtinctEDII ~ 1,                family = quasi(link ="logit", variance = "mu(1-mu)"),data = D3)summary(nullModel)#Full modelCombinedMod3Q = glm(LargePropExtinctEDII ~ HumanCat7 + MatAnomStand +  PrecipVelStand + HumanCat7 * MatAnomStand +                      HumanCat7 *  PrecipVelStand,                    family = quasi(link ="logit", variance = "mu(1-mu)"),data = D3)summary(CombinedMod3Q)model1 = anova(nullModel,CombinedMod3Q,test="Chisq")#Removed Precip interactionCombinedNoPI = glm(LargePropExtinctEDII ~ HumanCat7 + MatAnomStand +  PrecipVelStand + HumanCat7 * MatAnomStand,                   family = quasi(link ="logit", variance = "mu(1-mu)"),data = D3)summary(CombinedNoPI)model2 = anova(CombinedNoPI,CombinedMod3Q,test="Chisq")#Removed Temp interactionCombinedNoTI = glm(LargePropExtinctEDII ~ HumanCat7 + MatAnomStand +  PrecipVelStand + HumanCat7 *  PrecipVelStand,                   family = quasi(link ="logit", variance = "mu(1-mu)"),data = D3)summary(CombinedNoTI)model3 = anova(CombinedNoTI,CombinedMod3Q,test="Chisq")#Removed Precip CombinedNoPrecip = glm(LargePropExtinctEDII ~ HumanCat7 + MatAnomStand + HumanCat7 * MatAnomStand,                       family = quasi(link ="logit", variance = "mu(1-mu)"),data = D3)summary(CombinedNoPrecip)model4 = anova(CombinedNoPrecip,CombinedMod3Q,test="Chisq")#Removed TempCombinedNoTemp = glm(LargePropExtinctEDII ~ HumanCat7 + PrecipVelStand + HumanCat7 *  PrecipVelStand,                     family = quasi(link ="logit", variance = "mu(1-mu)"),data = D3)summary(CombinedNoTemp)model5 = anova(CombinedNoTemp,CombinedMod3Q,test="Chisq")#Only TempOnlyTemp = glm(LargePropExtinctEDII ~ HumanCat7 + MatAnomStand,               family = quasi(link ="logit", variance = "mu(1-mu)"),data = D3)summary(OnlyTemp)model6 = anova(OnlyTemp,CombinedMod3Q,test="Chisq")#Only PrecipOnlyPrecip = glm(LargePropExtinctEDII ~ HumanCat7 + PrecipVelStand,                 family = quasi(link ="logit", variance = "mu(1-mu)"),data = D3)summary(OnlyPrecip)model7 = anova(OnlyPrecip,CombinedMod3Q,test="Chisq")#No ClimateNoClimate = glm(LargePropExtinctEDII ~ HumanCat7,                family = quasi(link ="logit", variance = "mu(1-mu)"),data = D3)summary(NoClimate)model8 = anova(NoClimate,CombinedMod3Q,test="Chisq")#No HumanNoHuman = glm(LargePropExtinctEDII ~ MatAnomStand +  PrecipVelStand,              family = quasi(link ="logit", variance = "mu(1-mu)"),data = D3)summary(NoHuman)model9 = anova(NoHuman,CombinedMod3Q,test="Chisq")model1model2model3model4model5model6model7model8model9#Full model is significantly better than all others# Calculate Pseudo- R2# Combined modelPredicted =  predict(CombinedMod3Q,data.frame(HumanCat7 = D3$HumanCat7,PrecipVelStand = D3$PrecipVelStand,MatAnomStand = D3$MatAnomStand),type = "response")Observed = D3$LargePropExtinctEDIIcorrelation =cor(Predicted,Observed)correlation^2 # 0.7150543plot(Observed,Predicted)# Human onlyPredicted =  predict(NoClimate,data.frame(HumanCat7 = D3$HumanCat7),type = "response")Observed = D3$LargePropExtinctEDIIcorrelation =cor(Predicted,Observed)correlation^2 # 0.629plot(Observed,Predicted)# Climate onlyPredicted =  predict(NoHuman,data.frame(PrecipVelStand = D3$PrecipVelStand,MatAnomStand = D3$MatAnomStand),type = "response")Observed = D3$LargePropExtinctEDIIcorrelation =cor(Predicted,Observed)correlation^2 # 0.201plot(Observed,Predicted)########################################### SAR Analysis###########################################Perform arcsine sqrt transformationD3$LargePropExtinctEDIIArcSine = trans.arcsine(D3$LargePropExtinctEDII)########################################### Correlations with Dutilleul correction for SAC##########################################modified.ttest(D3$LargePropExtinctEDIIArcSine, D3$MatAnomStand, coords = as.matrix(cbind(D3$X,D3$Y)), nclass = NULL)#Corrected Pearson's correlation for spatial autocorrelation#data: x and y ; coordinates:  #F-statistic: 2.5796 on 1 and 12.4006 DF, p-value: 0.1334 #alternative hypothesis: true autocorrelation is not equal to 0#sample correlation: 0.415modified.ttest(D3$LargePropExtinctEDIIArcSine, D3$PrecipAnomStand, coords = as.matrix(cbind(D3$X,D3$Y)), nclass = NULL)#Corrected Pearson's correlation for spatial autocorrelation#data: x and y ; coordinates:  #F-statistic: 0.724 on 1 and 44.3244 DF, p-value: 0.3994  #alternative hypothesis: true autocorrelation is not equal to 0#sample correlation: -0.1268modified.ttest(D3$LargePropExtinctEDIIArcSine, D3$TempVelStand, coords = as.matrix(cbind(D3$X,D3$Y)), nclass = NULL)#Corrected Pearson's correlation for spatial autocorrelation#data: x and y ; coordinates:  #F-statistic: 1.7913 on 1 and 15.5978 DF, p-value: 0.1999 #alternative hypothesis: true autocorrelation is not equal to 0#sample correlation: 0.321modified.ttest(D3$LargePropExtinctEDIIArcSine, D3$PrecipVelStand, coords = as.matrix(cbind(D3$X,D3$Y)), nclass = NULL)#Corrected Pearson's correlation for spatial autocorrelation#data: x and y ; coordinates:  #F-statistic: 0.1269 on 1 and 54.7304 DF, p-value: 0.723#alternative hypothesis: true autocorrelation is not equal to 0#sample correlation: -0.0481modified.ttest(D3$LargePropExtinctEDIIArcSine, as.numeric(D3$HumanCat7), coords = as.matrix(cbind(D3$X,D3$Y)), nclass = NULL)#Corrected Pearson's correlation for spatial autocorrelation#data: x and y ; coordinates:  #F-statistic: 8.1775 on 1 and 4.8575 DF, p-value: 0.0366 #alternative hypothesis: true autocorrelation is not equal to 0#sample correlation: 0.7921###########################################SAR###########################################Sets up the spatial matrix# Neighbourhood with 4 connectorscoords = as.matrix(cbind(D3$X, D3$Y))Neig = knn2nb(knearneigh(coords, 4, longlat = TRUE))W3 = nb2listw(Neig,style="W",zero.policy =T)# Start by doing the SAR for full model#SAR error WSAR3 = errorsarlm(D3$LargePropExtinctEDIIArcSine ~ D3$HumanCat7 +  D3$MatAnomStand + D3$PrecipVelStand + D3$HumanCat7 * D3$MatAnomStand + D3$HumanCat7 * D3$PrecipVelStand,listw=W3,zero.policy =T)summary(SAR3)corr3 = correlog(D3$X, D3$Y,SAR3$residuals, increment =500, resamp=0,latlon=T) plot(corr3)SAR3 = errorsarlm(D3$LargePropExtinctEDIIArcSine ~ D3$HumanCat7 +  D3$MatAnomStand + D3$PrecipVelStand + D3$HumanCat7 * D3$MatAnomStand + D3$HumanCat7 * D3$PrecipVelStand,listw=W3,zero.policy =T,data=D3)summary(SAR3)Predicted =  predict(SAR3,data.frame(HumanCat7 = D3$HumanCat7,MatAnomStand = D3$MatAnomStand, PrecipVelStand = D3$PrecipVelStand),type = "response")Observed = D3$LargePropExtinctEDIIArcSinecorrelation =cor(Predicted,Observed)correlation^2 #  0.6743081CombinedSAR = summary(SAR3)xmat = matrix(nrow = 9, ncol=2)xmat[1:9,1] = CombinedSAR$Coef[1:9]xmat[1:9,2] = CombinedSAR$rest.se[1:9]summary(SAR3)#Now reduce the full model and compare by AIC#No Precip interactionSARNPI3 = errorsarlm(D3$LargePropExtinctEDIIArcSine ~ D3$HumanCat7 +  D3$MatAnomStand + D3$PrecipVelStand + D3$HumanCat7 * D3$MatAnomStand,listw=W3,zero.policy =T,data=D3)summary(SARNPI3)#AIC = -268.62corr3 = correlog(D3$X, D3$Y,SARNPI3$residuals, increment =500, resamp=0,latlon=T) plot(corr3)#No Temp interactionSARNTI3 = errorsarlm(D3$LargePropExtinctEDIIArcSine ~ D3$HumanCat7 +  D3$MatAnomStand + D3$PrecipVelStand + D3$HumanCat7 * D3$PrecipVelStand,listw=W3,zero.policy =T,data=D3)summary(SARNTI3)#AIC = -265.91corr3 = correlog(D3$X, D3$Y,SARNTI3$residuals, increment =500, resamp=0,latlon=T) plot(corr3)#No PrecipSARNoPrecip3 = errorsarlm(D3$LargePropExtinctEDIIArcSine ~ D3$HumanCat7 +  D3$MatAnomStand + D3$HumanCat7 * D3$MatAnomStand,listw=W3,zero.policy =T)summary(SARNoPrecip3)#AIC = -270.53corr3 = correlog(D3$X, D3$Y,SARNoPrecip3$residuals, increment =500, resamp=0,latlon=T) plot(corr3)#No TemperatureSARNoTemp3 = errorsarlm(D3$LargePropExtinctEDIIArcSine ~ D3$HumanCat7 + D3$PrecipVelStand + D3$HumanCat7 * D3$PrecipVelStand,listw=W3,zero.policy =T)summary(SARNoTemp3)#AIC = -266.8corr3 = correlog(D3$X, D3$Y,SARNoTemp3$residuals, increment =500, resamp=0,latlon=T) plot(corr3)#No HumanSARNoHuman3 = errorsarlm(D3$LargePropExtinctEDIIArcSine ~  D3$MatAnomStand + D3$PrecipVelStand,listw=W3,zero.policy =T)summary(SARNoHuman3)#AIC = -245.39corr3 = correlog(D3$X, D3$Y,SARNoHuman3$residuals, increment =500, resamp=0,latlon=T) plot(corr3)#No ClimateSAR3NoClimate = errorsarlm(D3$LargePropExtinctEDIIArcSine ~ D3$HumanCat7 ,listw=W3,zero.policy =T)summary(SAR3NoClimate)#AIC =-269.01SAR3AICs = c(AIC(SAR3), AIC(SARNPI3), AIC(SARNTI3), AIC(SARNoPrecip3), AIC(SARNoTemp3), AIC(SARNoHuman3),AIC(SAR3NoClimate))min(SAR3AICs)# No precip is bestsummary(SARNoPrecip3)Predicted =  predict(SARNoPrecip3,data.frame(HumanCat7 = D3$HumanCat7,MatAnomStand = D3$MatAnomStand),type = "response")Observed = D3$LargePropExtinctEDIIArcSinecorrelation =cor(Predicted,Observed)correlation^2 #  0.6836569plot(Observed,Predicted)OLSArcFull = lm(LargePropExtinctEDIIArcSine ~ HumanCat7 * MatAnomStand, data=D3)summary(OLSArcFull)corrOLS = correlog(D3$X, D3$Y,OLSArcFull$residuals, increment =500, resamp=0,latlon=T) corr3 = correlog(D3$X, D3$Y,SARNoPrecip3$residuals, increment =500, resamp=0,latlon=T) plot(corrOLS)
